# A Toxicogenomic Comparison of Primary and Photochemically Altered Air Pollutant Mixtures

**DOI:** 10.1289/ehp.1003323

**Published:** 2011-07-14

**Authors:** Julia E. Rager, Kim Lichtveld, Seth Ebersviller, Lisa Smeester, Ilona Jaspers, Kenneth G. Sexton, Rebecca C. Fry

**Affiliations:** 1 Department of Environmental Sciences and Engineering, Gillings School of Global Public Health, University of North Carolina at Chapel Hill, Chapel Hill, North Carolina, USA; 2 Center for Environmental Medicine, Asthma, and Lung Biology, School of Medicine, University of North Carolina at Chapel Hill, Chapel Hill, North Carolina, USA

**Keywords:** air pollution, biomarkers, environment, gene regulation, lung cells, systems biology

## Abstract

Background: Air pollution contributes significantly to global increases in mortality, particularly within urban environments. Limited knowledge exists on the mechanisms underlying health effects resulting from exposure to pollutant mixtures similar to those occurring in ambient air. In order to clarify the mechanisms underlying exposure effects, toxicogenomic analyses are used to evaluate genomewide transcript responses and map these responses to molecular networks.

Objectives: We compared responses induced by exposure to primary pollutants and photochemically altered (PCA) pollutant mixtures representing urban atmospheres to test our hypothesis that exposures to PCA pollutants would show increased modulation of inflammation-associated genes and pathways relative to primary air pollutants.

Methods: We used an outdoor environmental irradiation chamber to expose human lung epithelial cells to mixtures representing either primary or PCA pollutants for 4 hr. Transcriptional changes were assessed using microarrays and confirmed using quantitative real-time reverse-transcription polymerase chain reaction (qRT-PCR) on a subset of genes.

Results: We found a large difference in the cellular responses to the two pollutant exposures: Primary air pollutants altered the expression levels of 19 genes, whereas PCA pollutants altered 709 genes. Functional and molecular analyses of the altered genes revealed novel pathways, such as hepatocyte nuclear factor 4α, potentially regulating the pollutant responses. Chemical component analysis characterized and confirmed the photochemical transformation of primary air pollutants into PCA air pollutants.

Conclusions: Our study shows that the photochemical transformation of primary air pollutants produces altered mixtures that cause significantly greater biological effects than the primary pollutants themselves. These findings suggest that studying individual air pollutants or primary pollutant mixtures may greatly underestimate the adverse health effects caused by air pollution.

Outdoor air pollution contributes significantly to global increases in mortality, particularly within urban environments (Brunekreef and Holgate 2002; Burnett et al. 2000). Some health effects of air pollution may occur over time from chronic exposure, as with bronchitis and lung cancer (Brunekreef and Holgate 2002). Other health effects occur more abruptly, as with acute respiratory symptoms, decreased lung function, and cardiovascular effects (Brunekreef and Holgate 2002). Despite these adverse health outcomes, the underlying molecular mechanisms associating air pollutant exposure with disease remain largely unknown. To contribute to the understanding of air-pollutant–induced health effects, we used an *in vitro* model system involving short-term air exposures to identify new cellular mechanisms potentially influencing air toxic responses.

To link air pollution exposure to health outcomes, it is important to accurately characterize exposure conditions by understanding air pollution chemistry. Within urban environments, primary pollutants such as nitrogen oxides (NO_x_) and volatile organic compounds (VOCs) are emitted into the atmosphere. These primary pollutants then react to generate various secondary products, including ozone, peroxyacetyl nitrate, formaldehyde, and other carbonyls [U.S. Environmental Protection Agency (EPA) 2006]. This complex atmospheric chemistry creates an environment that is constantly changing, making it difficult to understand the causes and mechanisms of air-pollutant–induced disease.

Our study builds on an investigation comparing human lung cell responses to different air pollutant mixtures (Sexton et al. 2004). That study showed significant increases in the release of inflammatory cytokines in lung cells exposed to photochemically altered (PCA) air pollutants. In the present study, we further investigated this response by implementing a toxicogenomic approach to compare the genomewide transcriptional profiles of lung cells exposed to primary emitted pollutant mixtures relative to PCA air mixtures. Toxicogenomic studies can be used to assess genomewide alterations in mRNA levels, providing information on the genes and biological pathways that are modified by environmental exposures (McHale et al. 2010). To our knowledge, this is the first study to use a toxicogenomic approach to compare the genomic changes resulting from primary and PCA air pollutant mixture exposures.

In this study we investigated the toxicogenomic response of lung cells exposed to gaseous mixtures that represent urban atmospheric conditions. Specifically, we analyzed the transcriptomic response of cells exposed to common air pollutants reacting photochemically with sunlight. By using an environmental irradiation chamber, we compared gene expression patterns and inflammatory responses in cells exposed to complex mixtures representing primary pollutants and PCA pollutants.

## Materials and Methods

*Generation of pollutant mixtures.* We used the University of North Carolina–Chapel Hill’s outdoor environmental irradiation chamber (120 m^3^ volume) to prepare exposure conditions. Outdoor environmental irradiation chambers are photochemical reactors that use sunlight to initiate the natural photochemical transformation chemistry of air pollutants (Jeffries 1995; Jeffries et al. 1976; Sexton et al. 2004). Synthetic Urban Mix (Scott Specialty Gases, Plumsteadville, PA), a VOC mixture, and NO_x_ [nitric oxide (NO) and nitrogen dioxide (NO_2_)] were used as the starting material for the test atmosphere. Synthetic Urban Mix contains 55 different hydrocarbons at specific ratios that represent chemicals present in urban atmospheres (Jeffries and Sexton 1995). On the morning of the exposure day, the chamber was naturally humidified by preflushing with HEPA-filtered ambient air. At 0715 hours, the volatile organics of Synthetic Urban Mix were drawn from a gas cylinder into the environmental irradiation chamber while a liquid mixture containing less-volatile organics was injected into the chamber. In addition, NO_x_ was drawn from a gas cylinder (AirGas National Welders, Morrisville, NC) into the chamber to establish a test atmosphere containing 2 ppm carbon Synthetic Urban Mix and 0.2 ppm NO_x_. This test atmosphere remained inside the environmental irradiation chamber throughout the day, where sunlight induced photochemical reactions among the mixture’s compounds and secondary products were generated.

*Cell culture.* Human A549 type II lung epithelial cells, derived from a human lung adenocarcinoma, were cultured according to standard protocol (ATCC, Manassas, VA). Cells were grown as described previously (Jaspers et al. 1997; Sexton et al. 2004) and plated onto 24-mm-diameter collagen-coated membranes with 0.4-μm pores (Trans-CLR; Costar, Cambridge, MA). Immediately before exposure, media were removed to create direct air–liquid interface culture conditions, as previously described (Jaspers et al. 1997; Sexton et al. 2004).

*Exposure to pollutant mixtures.* We used a coupled chamber–*in vitro* exposure system for this study (Sexton et al. 2004). Sample lines directly linked the environmental irradiation chamber’s mixture to a cellular exposure chamber (Modular Incubator Chamber; Billups-Rothenberg, Del Mar, CA), where air exposures were continuously drawn through at 1.0 L/min. The 8-L cellular exposure chamber was positioned within an incubator (set at 37°C), where CO_2_ was added to the exposure source stream at 0.05 L/min, and a small water dish provided humidification to maintain cell viability and match control conditions.

The first exposure was in the morning, representing the primary pollutant mixture exposure. Lung cells were exposed for 4 hr (0815–1215 hours), and time-matched control cells were exposed to incubator air. The second exposure was in the evening, representing the PCA pollutant mixture, which contained primary pollutants and secondary products of irradiation chemistry. For this treatment, prepared lung cells were exposed for 4 hr (1630–2030 hours), and another set of mock-treated control cells were exposed to incubator air. For each exposure period, 4 exposed and 4 unexposed samples were used, resulting in a total of 16 total samples. Cells were incubated for 9 hr after each respective exposure period. Cells were then scraped and stored at –80°C in TRIzol_®_ Reagent (Invitrogen Life Technologies, Carlsbad, CA), and supernatants were aspirated and stored at –80°C.

*Chemical analysis of atmospheric mixtures.* We used gas measurement methods to assess the chemical constituents inside the chamber during the experiment. Volatile organic hydrocarbon levels were measured five times during the experiment using a Varian STAR 3400 DB-1 capillary gas chromatograph-flame ionization detection (Scientific Instruments, Cary, NC) with a Varian Saturn 2000 ion trap mass spectrometer. Ozone was measured every minute using a U.S. EPA equivalent method (EQOA-0193-091; U.S. EPA. 2011) based on ultraviolet photometry with a Teledyne model 9811 monitor (Teledyne Monitor Labs, Englewood, CO). NO and NO_2_ levels were measured every minute using a U.S. EPA standard reference method (RFNA-1292-090; U.S. EPA. 2011) based on chemiluminescence with a Teledyne model 9841 NO_x_ analyzer. Formaldehyde concentrations were measured continuously using a Dasgupta diffusion-tube sampler (Dasgupta et al. 1988). Peroxyacetyl nitrate levels were measured every 30 min using a Varian CP-3800 gas chromatograph with an electron capture detector. A scanning mobility particle sizer, with a model 3080 electrostatic classifier, and a model 3025a ultrafine condensation particle counter (both from TSI Inc., Shoreview, MN) were used to detect possible particulate matter formation.

*Analysis of cytotoxicity.* To measure cytotoxicity, we measured the enzyme lactate dehydrogenase (LDH) within the supernatant of each sample using a coupled enzymatic assay (Clontech Laboratories, Inc., Mountain View, CA) according to the manufacturer’s instructions. For each exposure period, four unexposed and four exposed sample wells were analyzed in technical triplicate for a total of 24 measurements. Scanned absorbance reading outliers were identified through the Grubbs’ test, where outliers were identified as those with < 5% probability of occurring as an outlier by chance alone, relative to a normal distribution (Grubbs 1969). Fold increase was calculated by dividing the mean LDH level of exposed cells by that of unexposed cells. Statistical significance of LDH levels in the exposed versus unexposed cells, as well as the PCA-pollutant–induced versus primary-pollutant–induced LDH levels, was calculated using an unpaired *t*-test with Welch’s correction (Welch 1938).

*Microarray processing.* Total RNA was isolated from unexposed and exposed cells using an RNeasy_®_ Mini Kit (Qiagen, Valencia, CA) according to the manufacturer’s protocol. RNA was quantified with the NanoDrop™ 1000 Spectrophotometer (Thermo Scientific, Waltham, MA), and its integrity was verified with a model 2100 bioanalyzer (Agilent Technologies, Santa Clara, CA). RNA was biotin labeled according to the Affymetrix protocol and hybridized to Affymetrix GeneChip_®_ Human Gene 1.0 ST arrays (Affymetrix Inc., Santa Clara, CA), which probe for 28,869 genes. Samples were assessed in biological duplicate for each exposure condition—primary pollutant exposures and time-matched unexposed controls, and PCA pollutant exposures and time-matched unexposed controls—for a total of eight microarray samples.

*Microarray analysis.* Microarray data were first normalized using robust multichip average (Irizarry et al. 2003). Genes with signal intensities < 30 (10% of the average signal intensity) in all conditions were removed from the analysis to eliminate background noise. This resulted in a reduction of probe sets from 28,869 for each condition to 24,652 for primary pollutant conditions and 24,830 for PCA pollutant conditions. Differential gene expression was defined as a significant difference in mRNA levels between exposed versus unexposed samples, where the following three statistical requirements were set: *a*) fold change of ≥ 1.5 or ≤ –1.5 (exposed vs. unexposed); *b*) *p* < 0.05 [analysis of variance (ANOVA)]; and *c*) a false discovery rate corrected *q-*value < 0.05. ANOVA *p*-values were calculated using Partek_®_ Genomics Suite_™_ software (Partek, Inc., St. Louis, MO). To control the rate of false positives, *q*-values were calculated as the minimum positive false discovery rate that can occur when identifying significant hypotheses (Storey 2003). Microarray data have been submitted to the National Center for Biotechnology Information (NCBI) Gene Expression Omnibus repository (Edgar et al. 2002) and are available under accession no. GSE23735 (NCBI 2010).

*Quantitative reverse-transcription polymerase chain reaction (RT-PCR) verification of mRNA expression.* Expression levels of genes selected based on magnitudes of toxicant-induced fold changes were validated using quantitative real-time RT-PCR (qRT-PCR). We used QuantiTect Primer Assays (Qiagen) for glutamine-fructose-6-phosphate transaminase 2 (*GFPT2*; catalog no. QT00007854), 2´,5´-oligoadenylate synthetase 1 (*OAS1*; catalog no. 00099134), and ATPase, class I, type 8B, member 1 (*ATP8B1*; catalog no. QT00038094) with the Qiagen QuantiTect SYBR_®_ Green PCR kit and Roche Lightcycler 480 (Roche Diagnostics Corp., Indianapolis, IN). Fold changes between exposed and unexposed samples were calculated based on cycle time values and normalized to the β-actin housekeeping gene. Statistical significance of the exposed versus unexposed transcript levels, as well as the primary versus PCA-induced transcript changes, was calculated using an unpaired *t*-test.

*Network analysis and enriched biological functions.* Molecular networks and biological functions associated with air pollutant exposure were identified using the Ingenuity database (Ingenuity_®_ Systems, Redwood City, CA). The Ingenuity database provides a collection of gene-to-phenotype associations, molecular interactions, regulatory events, and chemical knowledge accumulated to develop a global molecular network. The lists of differentially expressed genes were overlaid onto this global molecular network, where related networks were algorithmically constructed based on connectivity. We evaluated significance for each constructed network using the following equation, based on Fisher’s exact test (Calvano et al. 2005):


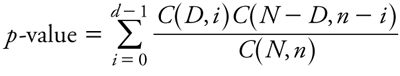
[1]

Here, *N* is the total number of molecules in Ingenuity’s global network, which contains *D* total proteins encoded by differentially expressed genes. Each individual network contains *n* molecules, *d* of which are proteins encoded by differentially expressed genes (Calvano et al. 2005). The *p*-value is the summation of each fraction, calculated across all *d*s, represented by *i* = 0, 1, 2, 3, . . ., *d* – 1. *C* is the binomial coefficient, calculated using the following equation (Daniel 2009):

*C*(*N*,*n*) = *N*! ÷ [*n*!(*N!* – *n)*!]. [2]

Only networks with *p* < 10^–12^ were evaluated.

We performed functional analysis to identify biological functions and disease signatures significantly associated with the differentially expressed genes. Statistical significance of each biological function or disease was calculated using a right-tailed Fisher’s exact test. Functions with *p* ≤ 3.0 × 10^–6^ were considered significant.

*Transcription factor binding site analysis.* We performed transcription factor binding site analysis using EXPANDER software (version 5.1; Algorithms in Computational Genomics at Tau 2010). Affymetrix probe sets were linked to sequence data in regions 1,000 base pairs upstream and 200 base pairs downstream of the transcription start sites. These sequence data were then analyzed for significant enrichment of transcription factor binding site sequences. *p*-Values represent the probability of obtaining an equal or greater number of matched binding site sequences using a randomly drawn sample of the same size as the input sequence set.

*Inflammatory cytokine release.* Protein levels of the cytokine interleukin (IL)-8 were measured using the supernatant samples. An OptEIA™ human IL-8 enzyme-linked immunosorbent assay (ELISA) (BD Biosciences, San Jose, CA) was performed and analyzed according to the manufacturer’s protocol. Scanned absorbance-reading outliers were identified, and statistical significance was calculated using the same method as for the LDH analysis. The fold increase of IL-8 was calculated by dividing mean IL-8 levels of exposed cells by those of unexposed cells.

## Results

**Figure 1:**
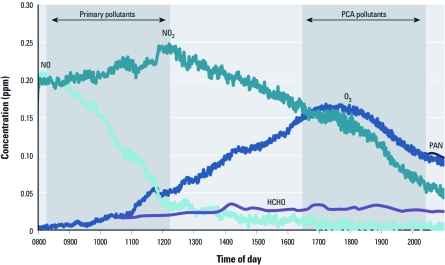
Chemical component analysis of the chamber exposure conditions showing levels of NO, NO_2_, ozone (O_3_), formaldehyde (HCHO), and peroxyacetyl nitrate (PAN).

*Chemical analysis of exposure conditions.* We exposed human lung cells to mixtures representing either primary air pollutants or PCA air pollutants, which contain secondary chemical products. To assess the gas composition of the exposures, we measured concentrations of > 40 reactive chemical species within the environmental irradiation chamber throughout the exposure periods. Average NO_2_ and NO levels decreased across the day, whereas levels of secondary chemical products, such as ozone, formaldehyde, and peroxyacetyl nitrate, increased (Table 1, Figure 1). Levels of most hydrocarbons decreased throughout the experiment (e.g., the aromatic compound toluene). More stable hydrocarbons, including *n*-hexane and benzene, remained stable across both exposures. No particulate matter or secondary organic aerosol was formed within the chamber (data not shown). For levels of all VOCs detected during both exposure periods, see Supplemental Material, Table 1 (http://dx.doi.org/10.1289/ehp.1003323).

**Table 1:** Average concentrations (ppb) of compounds measured throughout the exposure periods.

Pollutant exposure		
Chemical	Primary	PCA
Ozone		22		141
NO		130		6
NO_2_		213		119
Formaldehyde		18		29
Peroxyacetyl nitrate		0		62
Hydrocarbons (ppb carbon)				
Alkanes		872		378
*n*-Hexane		15		15
Alkenes		76		8
1-Octene		11		0
Aromatics		451		193
Benzene		35		31
Toluene		121		73
Exposure periods: primary pollutants, 0815–1215 hours; PCA pollutants, 1630–2030 hours. Examples are listed for each hydrocarbon group. For the complete hydrocarbon listing, see Supplemental Material, Table 1 (http://dx.doi.org/10.1289/ehp.1003323).				

It is important to recognize that the primary pollutant exposure did not contain completely fresh, unreacted compounds. As measured in the chemical analysis (Figure 1), some of the starting chemicals reacted throughout the primary pollutant exposure period. However, most of the photochemical aging reactions occurred during the afternoon, because outdoor conditions were cloudy until approximately 1200 hours. For simplicity, we refer to the morning exposure as the primary pollutant exposure and the afternoon exposure as the PCA pollutant exposure, recognizing that it is impossible for laboratory-generated mixtures to simulate these exposure conditions precisely.

*Cytotoxicity measurements.* We measured the release of LDH from lung cells after exposure. Lung cell death was greater after exposure to PCA pollutants (LDH fold increase = 9, *p* < 0.001) than after exposure to primary pollutants (LDH fold increase = 1.6, *p* < 0.07). The difference in LDH fold increase between the PCA pollutant–induced fold increase in LDH and the primary pollutant–induced fold increase in LDH was significant (*p* < 0.001).

*Gene expression analysis.* Lung cells were exposed to two different gaseous conditions: primary air pollutants in the morning and PCA pollutants in the afternoon. Each exposure condition was performed alongside untreated control samples. After exposure, we extracted mRNA from the cells and assessed relative transcript abundance using Affymetrix GeneChip_®_ Human Gene 1.0 ST microarrays.

**Figure 2:**
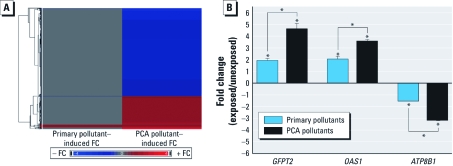
Air pollutant mixtures alter gene expression profiles in human lung cells. (*A*) Heat map showing average gene expression fold change (FC) of 714 total genes modulated by exposure to primary pollutant and/or PCA pollutant mixture. (*B*) qRT-PCR results displaying FC gene expression (mean ± SE). *p < 0.05 compared with the control or the other exposure condition.

Lung cells exposed to the primary pollutant mixture showed differential expression of 19 genes, 15 with increased expression and 4 with decreased expression levels (Figure 2A). In contrast, the PCA pollutant exposure caused changes in the expression levels of 709 genes. Of these genes, 190 showed increased expression levels and 519 showed decreased levels (Figure 2A). Among these two lists, 14 overlapping genes were differentially expressed in response to both pollutant mixtures [for a complete listing of genes with significant changes in expression for the two exposure conditions, see Supplemental Material, Table 2 (http://dx.doi.org/10.1289/ehp.1003323)]. Expression levels changed to a greater extent after PCA pollutant exposure than after primary pollutant exposure for 13 of the 14 overlapping genes. The extreme difference in the number of genes differentially expressed in response to PCA versus primary pollutant exposures persisted when parameters used to filter the data were altered (minimum fold change set to ± 1.5 and then required ANOVA *p*-values set to < 0.2 to < 0.0001) in a sensitivity analysis (data not shown).

*Validation of expression changes through qRT-PCR.* We used qRT-PCR to validate three genes selected based on their exposure-induced fold change magnitudes. From microarray analysis, *GFPT2* and *OAS1* were significantly up-regulated in expression and *ATP8B1* was significantly down-regulated by both primary and PCA pollutant exposures [see Supplemental Material, Table 2 (http://dx.doi.org/10.1289/ehp.1003323)]. PCR analysis confirmed significant (*p* < 0.05) up-regulation by air pollutant exposure in *GFPT2* (fold change = 1.85 and 4.52 for primary pollutants and PCA pollutants, respectively) and *OAS1* (fold change = 1.95 and 3.54; Figure 2B). PCR analysis also confirmed significant (*p* < 0.05) down-regulation of *ATP8B1* (fold change = –1.46 and –3.12).Furthermore, PCR analysis confirmed that these genes showed significantly (*p* < 0.05) greater fold change after exposure to PCA pollutants relative to primary pollutants.

**Figure 3:**
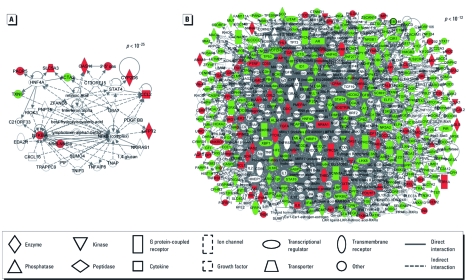
Network analysis of the genes affected by air pollutant exposure. Protein networks display the network associated with primary pollutant exposure (*A*) and the large interactome associated with PCA pollutant exposure (*B*). Symbols represent protein products of genes that are up-regulated (red), down-regulated (green), or associated with the differentially expressed genes (open).

*Network analysis and biological functions.* To identify potential biological pathways affected by air pollutant exposure in lung cells, the gene lists were overlaid onto molecular network maps. We algorithmically constructed networks containing the identified genes based on connectivity and known relationships among molecules. The 19 genes with altered expression from primary pollutant exposure generated one significant network (*p* < 10^–25^; Figure 3). This network consists of 35 total proteins, 9 of which are encoded by genes differentially expressed after primary pollutant exposure [see Supplemental Material, Table 3 (http://dx.doi.org/10.1289/ehp.1003323)]. Within this network, proteins that are associated with hepatocyte nuclear factor 4α (HNF4α) signaling showed altered expression levels (see Supplemental Material, Figure 1). There are also gene products related to cancer, respiratory disease, and inflammation, such as chemokine (C-C motif) ligand 2 (*CCL2*; also known as *MCP 1*).

**Figure 4:**
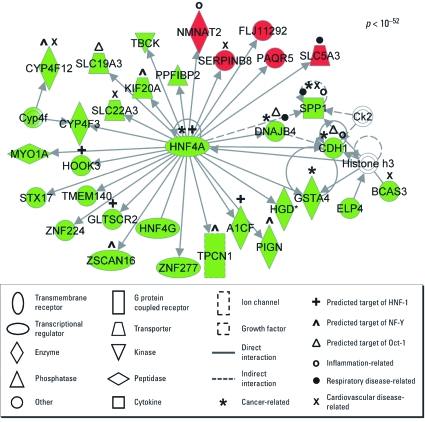
HNF4α signaling is altered by PCA pollutant exposure, as shown by the most significant network within the PCA-pollutant–associated interactome. Symbols represent protein products of up-regulated genes (red) and down-regulated genes (green). NF‑Y, nuclear transcription factor Y.

Overlaying the 709 PCA-pollutant–altered genes resulted in the generation of 25 significant networks [*p-*values ranging from 10^–12^ to 10^–52^; see Supplemental Material, Table 3 (http://dx.doi.org/10.1289/ehp.1003323)]. These 25 networks consist of 838 total proteins, 458 of which are encoded by genes differentially expressed after exposure to PCA pollutants Interestingly, we identified a large interacting protein network (i.e., interactome) containing 23 of the 25 networks modulated by PCA pollutants (*p* < 10^–12^; Figure 3). Within this interactome, we identified a highly significant (*p* < 10^–52^) smaller network (Figure 4) showing enrichment for the HNF4α pathway. As an alternative approach to the analyses, we used g:Profiler (Bioinformatics, Algorithmics and Data Mining Group 2011), which interprets gene lists by identifying enriched protein–protein interactions using the BioGRID database (BioGRID 2011). We also found the enrichment for proteins involved in HNF4α signaling using this alternative method (see Supplemental Material, Figure 2). Another network identified within the PCA-induced interactome shows a significant enrichment of proteins involved in inflammation linked to IL-8 and activator protein-1 (AP-1) signaling (*p* < 10^–27^) (see Supplemental Material, Figure 3). These subnetworks contain proteins known to be related to cancer, inflammation, cardiovascular disease, and respiratory disease.

To further understand the biological effects of air pollutant exposure, we queried the constructed networks for biological processes and disease signatures. The network associated with primary pollutant exposure showed significant association with one disease signature—cancer [*p* = 3 × 10^–6^; see Supplemental Material, Figure 4 (http://dx.doi.org/10.1289/ehp.1003323)]. PCA pollutant exposure, on the other hand, modified the expression of genes whose protein products are associated with 10 different biological processes (see Supplemental Material, Figure 4) including cancer (*p* < 1.9 × 10^–12^), cellular movement (*p* < 7.0 × 10^–10^), cellular growth and proliferation (*p* < 4.2 × 10^–7^), tissue development (*p* < 1.5 × 10^–6^), and cardiovascular disease (*p* < 1.8 × 10^–6^). As an alternative approach, we also performed a disease signature enrichment analysis using the functional annotation tool within the Database for Annotation, Visualization, and Integrated Discovery (DAVID) (Huang et al. 2009). Similar disease categories were identified as enriched using this alternate database (see Supplemental Material, Table 4).

*Transcription factors predicted to regulate response.* We performed transcription factor binding site analysis to predict regulatory mechanisms that potentially underlie gene expression changes associated with air pollution exposure. For the primary pollutants, analysis of the promoter regions of the 19 differentially expressed genes identified significant (*p* < 0.05) enrichment for binding sites of 17 transcription factors [see Supplemental Material, Table 5 (http://dx.doi.org/10.1289/ehp.1003323)]. In the PCA pollutant gene set, 53 transcription factors with significantly (*p* < 0.05) enriched binding sites were predicted (see Supplemental Material, Table 5). Comparison of the transcription factors associated with primary and PCA pollutant exposure revealed six common, overlapping transcription factors predicted to regulate the genomic responses to both exposure conditions. All six of these transcription factors are associated with genes that showed down-regulation upon exposure to air pollutants. The overlapping transcription factors with the most significant *p*-values were hepatocyte nuclear factor 1 (HNF1; *p* = 0.003), nuclear transcription factor Y(NF-Y; *p* = 0.005), and POU class 2 homeobox 1 (Oct-1; *p* = 0.014). All predicted targets are included in Supplemental Material, Table 5.

*Inflammatory cytokine IL-8 release.* Analysis showed that only a small amount of IL-8 was released from lung cells exposed to primary pollutants (average fold increase = 1.14; *p* = 0.50) compared with IL-8 release after exposure to PCA pollutants (average fold increase = 3.79; *p* < 0.001).

*Conservation of genes modified from air toxics exposures.* To gain further insight into the genes dysregulated by air toxics exposure, we compared our results with those of an existing genomic database from a study analyzing human lung epithelial cells exposed to cigarette smoke (Maunders et al. 2007). We performed this analysis because many chemicals are present in both cigarette smoke and PCA pollutant exposures, including benzene, formaldehyde, toluene, NO_x_, and various other hydrocarbons (U.S. EPA 1992). In the cigarette smoke study (Maunders et al. 2007), microarray analysis was performed to identify differentially expressed genes in lung cells at 1, 6, and 24 hr postexposure. In the present study, we identified a total of 52 genes with differential expression associated with cigarette smoke exposure (across all three time points) and PCA pollutants, 32 of which are down-regulated in all conditions [see Supplemental Material, Table 6 (http://dx.doi.org/10.1289/ehp.1003323)]. We used molecular network analysis to analyze the list of 32 air-toxics–modulated genes. The most significant (*p* < 10^–25^) network is enriched for the HNF4α signaling pathway (see Supplemental Material, Figure 5).

## Discussion

In this study, we compared the expression levels of > 25,000 genes in human lung epithelial cells exposed to mixtures representing either primary or PCA air pollutants. The complex air pollutant mixtures were generated in an outdoor environmental irradiation chamber simulating urban exposure conditions. The primary pollutant mixture contained compounds in fresh emissions at the early stages of atmospheric chemistry. In contrast, the PCA pollutant mixture consisted of compounds humans are exposed to in photochemically aged outdoor urban environments, with higher concentrations of reaction products formed from primary pollutants reacting throughout the day (Jeffries and Sexton 1995; Sexton et al. 2004).

Chemical component analysis of 43 compounds verified that the pollution chemistry analyzed within the environmental irradiation chamber was similar to urban air photochemistry (Jeffries and Sexton 1995). More specifically, levels of primary air pollutants, including NO_x_ and multiple VOCs, decreased throughout the experiment and were lower in the PCA pollutant mixture. At the same time, chemical reactions among NO_x_, VOCs, and sunlight generated secondary products present at higher concentrations in the PCA mixture, including ozone, formaldehyde, and peroxyacetyl nitrate. In general, the chemical concentrations within the PCA pollutant exposure were higher than most ambient levels but still comparable (Jeffries and Sexton 1995). For example, the average ozone concentration during the PCA exposure was approximately 140 ppb. Although this ozone level is considered high for most areas, concentrations ≥ 120 ppb have frequently been measured in certain cities as daily 1-hr maximum concentration levels (U.S. EPA 2006). Although we measured > 40 compounds, other chemicals in addition to those directly measured likely contributed to the observed cellular responses. Clearly, a complete chemical characterization of all chemicals was not possible.

In our study, we performed complex air chemistry exposures using an *in vitro* exposure system that allows direct exposures between chemically and physically unaltered air pollutants and target cells. We recognize that this setup does not completely mimic the *in vivo* human respiratory system. For example, *in vivo* the fluid layer coating lung epithelial cells contains proteins and antioxidants (Cross et al. 1994) that may mitigate the toxicity of inhaled chemicals. In addition, multiple cell types within the respiratory system may interact and influence inhalation responses in ways we are currently unable to simulate. Although A549 cells have been proposed as a standardized model to study lung epithelium (Foster et al. 1998), they are tumorous cells that may respond differently than primary cell lines. Still, the experimental setup fulfills our aim to perform a novel baseline study that generates testable hypotheses to be pursued mechanistically.

We found through our genomics analysis that exposure to primary pollutants altered the expression of only 19 genes, whereas exposure to PCA pollutants altered the expression of 709 genes. In addition, there was a difference in cytotoxicity. However, the differences in gene expression were unlikely to be related to this differential cytotoxicity. In support of this, it has been shown in various eukaryotic cell types that extent of genomic change is not associated with cell death (Jelinsky et al. 2000; Tsukue et al. 2010).

To identify molecular pathways influenced by exposure to air pollutants, the differentially expressed genes were integrated with their protein products and queried for known interactions. A significant difference was evident between the number of altered networks associated with exposure to primary and PCA pollutants. These results suggest that exposure to secondary reactive species created through photochemical reactions induces greater changes in lung cell molecular network signaling than does exposure to primary emitted air pollutants.

As revealed through network analysis, inflammatory response pathways were modulated in lung cells exposed to air pollutants. For example, *CCL2*, *IL-8*, and *AP-1* expression levels were all increased. CCL2 is a chemoattractant protein that recruits monocytes to sites of infection and trauma, and it regulates monocyte, dendritic cell, memory T cell, and basophil infiltration during inflammation (Charo and Ransohoff 2006). Also, increased *IL-8* expression is a biomarker of air-pollutant–induced lung inflammation (Jaspers et al. 1997; Sexton et al. 2004) and is associated with inflammatory lung disease (Charo and Ransohoff 2006). We verified the increased expression of IL-8 protein, which showed significantly increased levels associated with PCA pollutant exposure. Network analysis showed that AP-1 may regulate the altered IL-8 response pathway. Interestingly, ozone, a secondary air pollutant produced within PCA mixtures, has been shown to activate AP-1, which potentially regulates ozone-induced IL-8 release (Jaspers et al. 1997). Our results are therefore consistent with other studies (Jaspers et al. 1997; Sexton et al. 2004) showing that air pollution exposures, such as those in urban environments, may influence lung cell function by altering the levels of gene transcripts and proteins associated with inflammatory response pathways.

In addition to changes in inflammation-related proteins, our research revealed changes in genomic responses associated with other diseases, including cancer, respiratory disease, and cardiovascular disease. Air particulate matter exposure has been clearly linked to cardiovascular events (Brook et al. 2004). More recent investigations have revealed that cardiovascular toxicity can also be triggered by gaseous air pollutants (Campen et al. 2010; Lund et al. 2007). Our network analysis illustrates that exposure to gaseous air pollutant mixtures modified the expression levels of genes that encode proteins associated with cardiopulmonary effects.

To analyze regulatory mechanisms underlying changes in gene expression resulting from air pollutant exposures, we computationally predicted putative mediating transcription factors. We identified six transcription factors as potential regulators of the observed transcriptional response to both primary and PCA pollutant exposures. These transcription factors include Oct-1 and HNF1, whose binding sites are enriched in genes with decreased expression after exposure. The transcription factor Oct-1 targets genes encoding proteins involved in cancer-related metabolism, such as proteins that contribute to decreased mitochondrial function and increased glycolysis rates (Kang et al. 2009). In addition, loss of Oct-1 can cause cells to become hypersensitive to genotoxic and oxidative stress agents (Kang et al. 2009). The most significant enrichment was for HNF1, which has a known association with inflammatory pathway signaling underlying coronary heart disease (Armendariz and Krauss 2009). The transcription factor HNF1 is also known to control *HNF4*α transcription (Hatzis and Talianidis 2001). In the present study, *HNF4*α was down-regulated by PCA pollutant exposure, and HNF4α signaling was enriched within networks associated with both primary and PCA pollutants. In addition, to gain further understanding of the cellular effects of air toxics, we compared our PCA pollutant results with those of an existing genomics database from a study that evaluated human lung cells exposed to cigarette smoke (Maunders et al. 2007). Interestingly, the overlapping genes were also enriched for the HNF4α pathway. This comparison shows that similar regulatory mechanisms may underlie cellular responses to diverse air toxics.

Currently, information relating HNF1 and HNF4α signaling with lung cell function is limited. It is noteworthy that a very recent study showed that HNF4α expression is evident in pulmonary adenocarcinoma tissues (Kunii et al. 2011). To our knowledge, our study is the first to identify links between the transcription factors HNF1 and HNF4α and air pollutant responses in lung epithelial cells.

We used two exposure atmospheres in our study, with each atmosphere containing a large number of chemical compounds. When inhaled, these compounds are known to influence lung function (e.g., ozone) (U.S. EPA 2006) and cause cancer (e.g., acetaldehyde, benzene, formaldehyde) (World Health Organization 2010). Because of the complexity of the exposure atmospheres, we could not establish which chemicals within the mixtures caused the observed cellular responses. As shown in our previous study using the same chemical starting materials (Sexton et al. 2004), numerous compounds in addition to ozone contribute to PCA pollutant responses. Future research will investigate which chemical constituents, or combinations thereof, may be driving the observed differences in genomic response.

## Conclusions

This study applied toxicogenomics-based analyses to air chemistry research to reveal potential biological mechanisms that may underlie health effects induced by air mixtures. The genomewide comparison highlights a significantly more robust response in lung cells exposed to PCA pollutants compared with primary pollutants. Mapping the genes affected by pollutant exposures to their encoded protein products and analyzing their biological functions revealed a relationship between air pollution exposure and pathways associated with IL-8, AP-1, and HNF4α. These findings suggest that PCA pollutants, to a greater extent than freshly emitted air pollutants, may significantly contribute to adverse health effects related to air pollution. With these findings, we suggest that air toxicology studies and pollution control policies need to increase focus on secondary products within PCA air pollutant mixtures in order to improve public health.

## Supplemental Material

(2 MB) PDF
