# Supplementary material for: A Toxicogenomic Comparison of Primary and Photochemically Altered Air Pollutant Mixtures
Source: Environ Health Perspect. 2011 Jul 14;119(11):1583–9. doi: 10.1289/ehp.1003323 (PMC3226493; doi:10.1289/ehp.1003323)
Supplement: (2 MB) PDF [file ehp.1003323.s001.pdf]

Supplemental Material

A Toxicogenomic Comparison of Primary and Photochemically Altered Air Pollutant Mixtures

Julia E. Rager[1], Kim Lichtveld[1], Seth Ebersviller[1], Lisa Smeester[1], Ilona Jaspers[2], Kenneth G. Sexton[1], and Rebecca C. Fry[1]

Authors' Affiliations:
[1] Department of Environmental Sciences and Engineering, Gillings School of Global Public Health, University of North Carolina, Chapel Hill, North Carolina, USA
[2] Center for Environmental Medicine, Asthma, and Lung Biology, School of Medicine, University of North Carolina, Chapel Hill, North Carolina, USA

Supplemental Material

TABLE OF CONTENTS

Supplemental Material, Figure 1: Primary pollutant-associated network with HNF4α signaling ... 3

Supplemental Material, Figure 2: Network interactions associated with PCA pollutants involving HNF4α, as identified through g:Profiler ... 4

Supplemental Material, Figure 3: IL-8 and AP-1 network associated with PCA pollutants ... 5

Supplemental Material, Figure 4: Biological functions significantly associated with primary and PCA pollutant exposure ... 6

Supplemental Material, Figure 5: A common molecular network modulated by exposure to cigarette smoke or photochemically altered (PCA) air pollutants ... 7

Supplemental Material, Table 1: Volatile organic compounds detected through gas chromatography throughout the experiment day ... 8

Supplemental Material, Table 2: Genes identified as significantly differentially expressed upon exposure to primary or photochemically altered (PCA) pollutants. ... 10

Supplemental Material, Table 3: Network proteins associated with exposure to (A) primary and (B) photochemically altered (PCA) pollutant mixtures ... 29

Supplemental Material, Table 4: Disease categories identified by DAVID analysis as enriched in cells exposed to (A) primary pollutants, and (B) PCA pollutants ... 32

Supplemental Material, Table 5: Transcription factors predicted to regulate genes modified upon exposure to (A) both primary and PCA pollutants, (B) primary pollutants, and (C) PCA pollutant mixtures ... 33

Supplemental Material, Table 6: Genes commonly differentially expressed upon exposure to photochemically altered (PCA) pollutants or cigarette smoke (CS) ... 43

REFERENCES ... 45

Supplemental Material, Figure 1

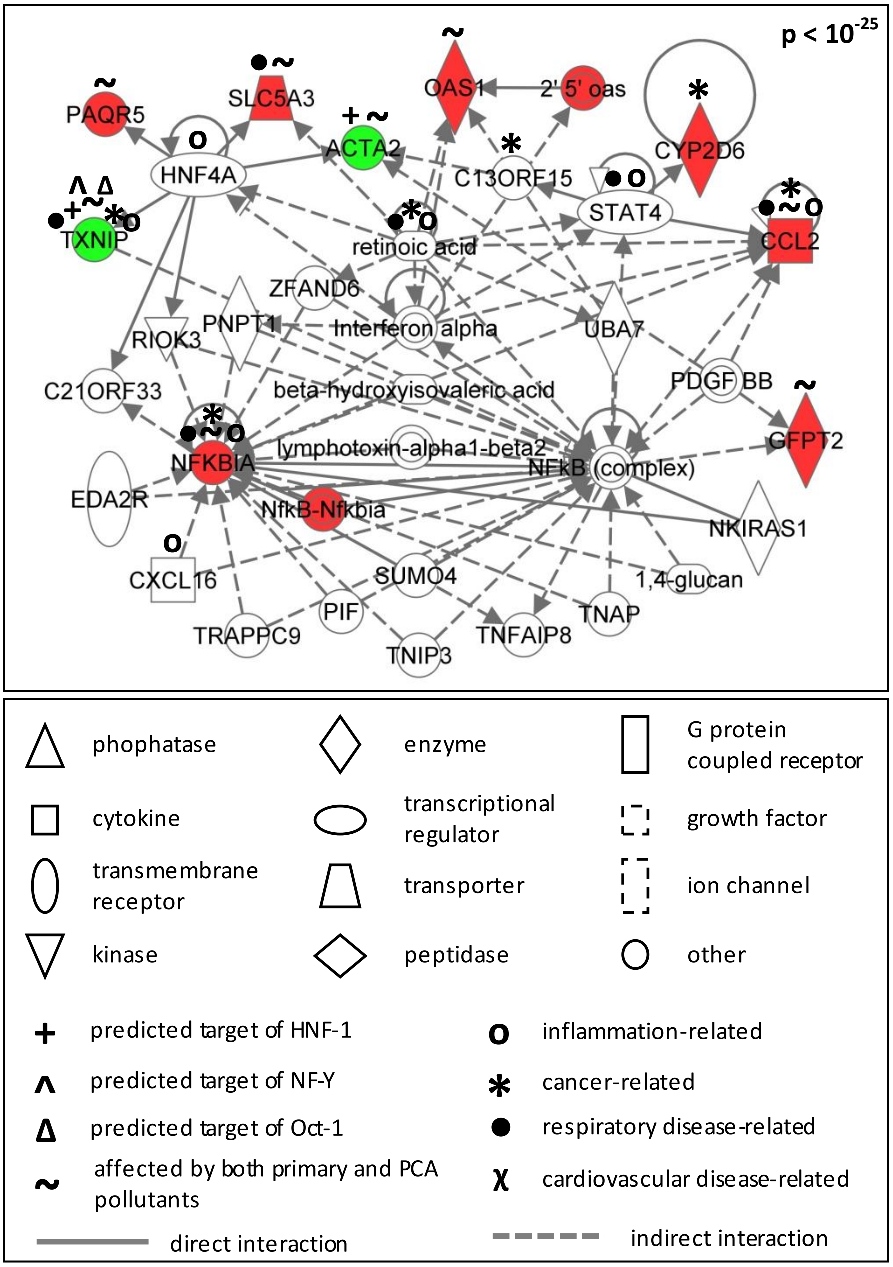

Supplemental Material, Figure 1: Primary pollutant-associated network with HNF4α signaling. Network is displayed with symbols representing protein products of genes that are up-regulated (red symbols), down-regulated (green symbols), or associated with the differentially expressed genes (clear symbols).

Supplemental Material, Figure 2

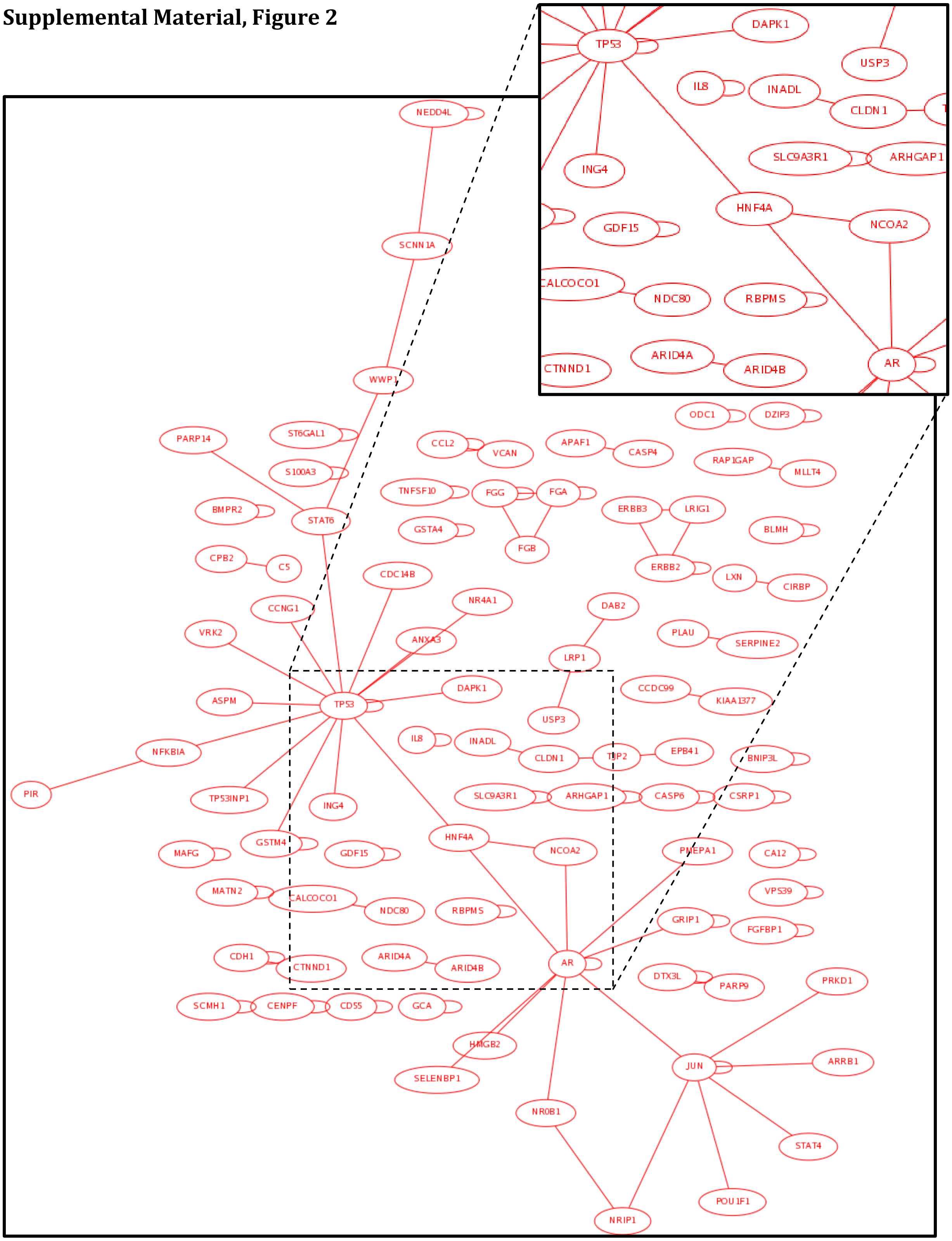

Supplemental Material, Figure 2: Network interactions associated with PCA pollutants involving HNF4α, as identified through g:Profiler. Protein-protein maps illustrate known interactions (as annotated in the BioGrid database) between proteins encoded by genes differentially expressed from PCA pollutant exposure (g:Profiler 2011, Reimand et al. 2007).

Supplemental Material, Figure 3

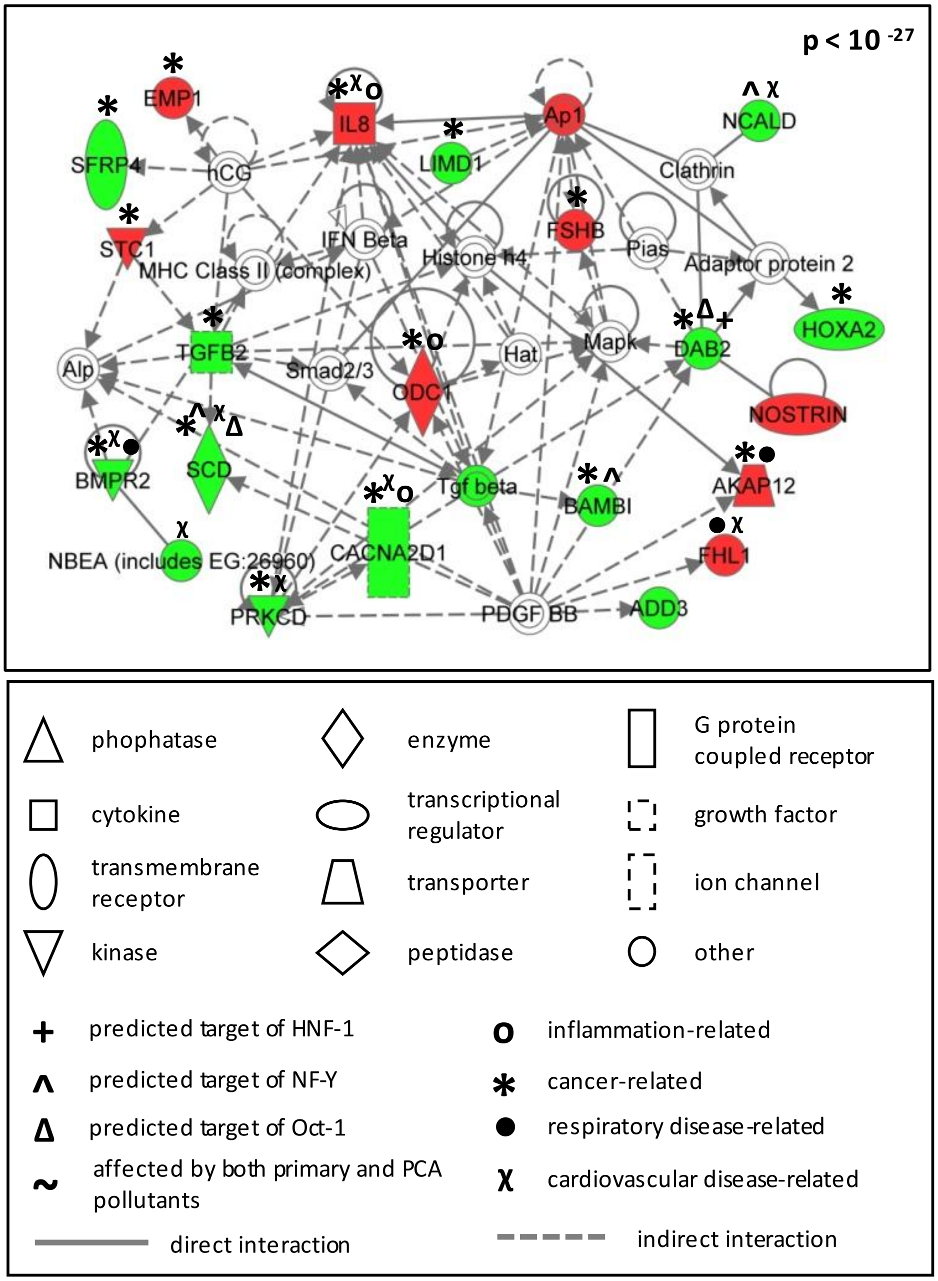

Supplemental Material, Figure 3: IL-8 and AP-1 network associated with PCA pollutants. Network is displayed with symbols representing protein products of genes that are up-regulated (red symbols), down-regulated (green symbols), or associated with the differentially expressed genes (clear symbols).

Supplemental Material, Figure 4

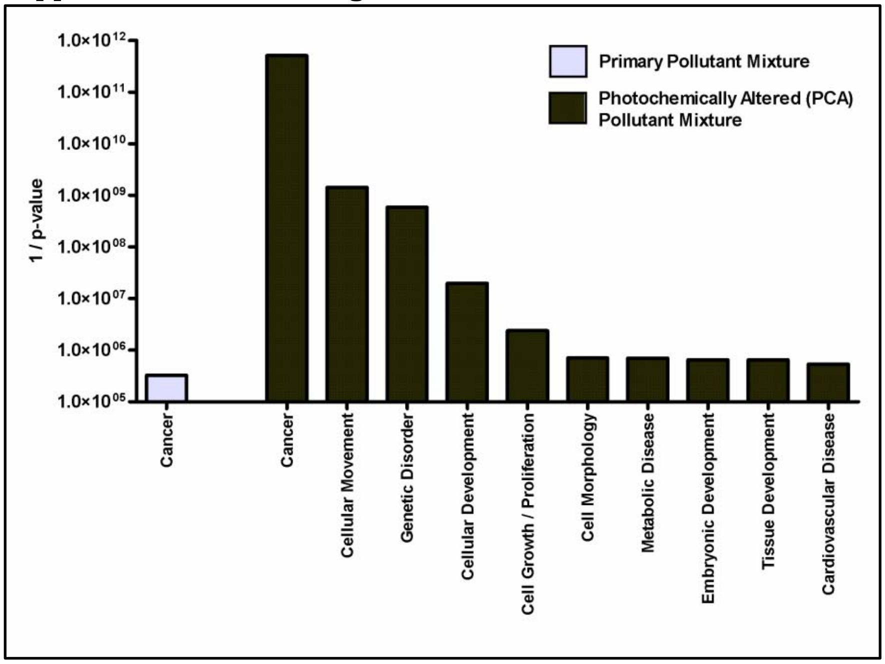

Supplemental Material, Figure 4: Biological functions significantly associated with primary and PCA pollutant exposure.

Supplemental Material, Figure 5

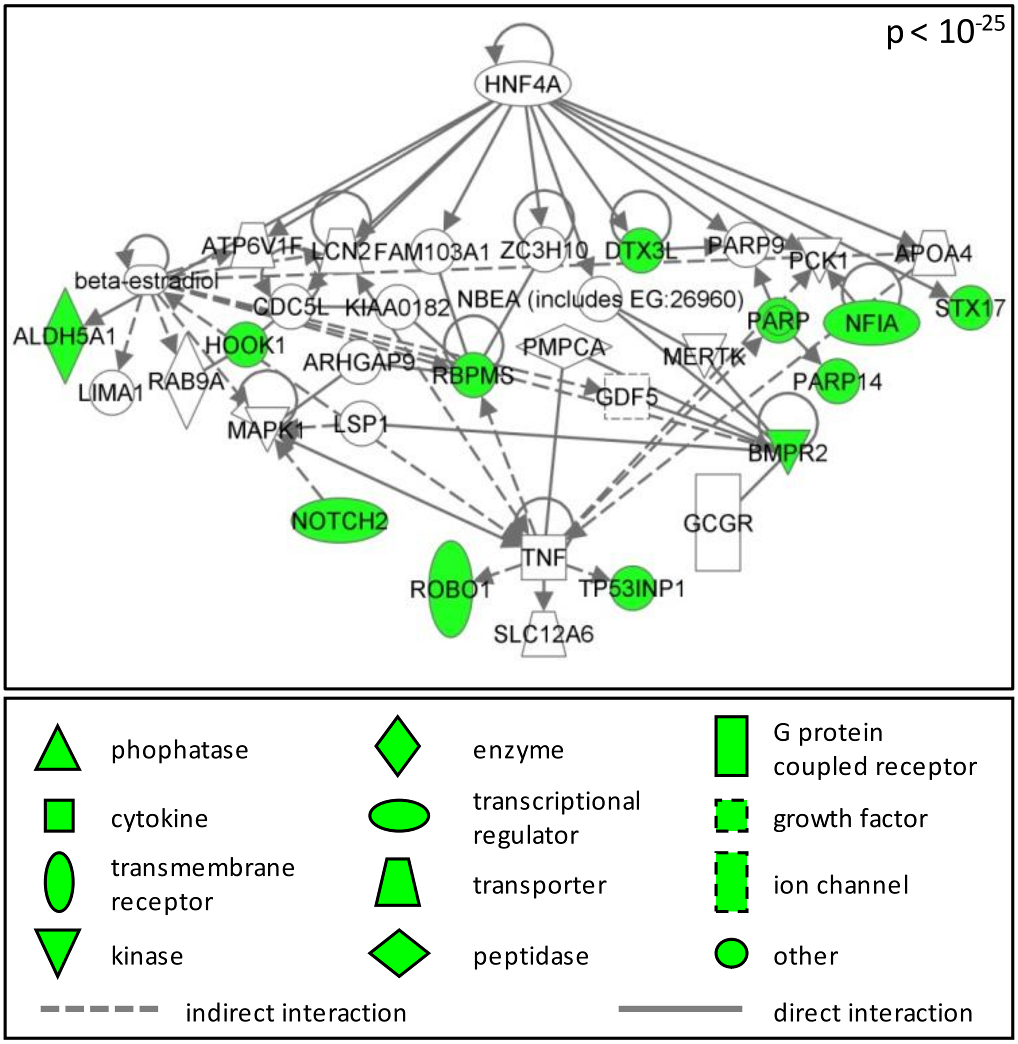

Supplemental Material, Figure 5: A common molecular network modulated by exposure to cigarette smoke or photochemically altered (PCA) air pollutants. This protein network displays the most significant network predicted to be altered in lung cells exposed to cigarette smoke (Maunders et al. 2007) or PCA pollutants. The network is displayed with symbols representing protein products of genes that down-regulated (green symbols) in response to both PCA and cigarette smoke, or associated with the differentially expressed genes (clear symbols).

Supplemental Material, Table 1: Volatile organic compounds detected through gas chromatography throughout the experiment day. Values are weighted averages taken from samples collected at 8:45 AM, 10:00 AM, 12:40 PM, 4:42 PM, and 5:57 PM.

Chemical	Chemical Group	Primary Pollutant Exposure (ppmC) (8:15 AM - 12:15 PM)	PCA Pollutant Exposure (ppmC) (4:30 PM - 8:30 PM)
**propane/propene**	85% alkane, 15% alkene	0.101	0.055
**isobutane**	alkane	0.035	0.026
**butane**	alkane	0.113	0.039
**isopentane**	alkane	0.137	0.040
**n-pentane**	alkane	0.086	0.022
**2-methylpentane**	alkane	0.033	0.019
**3-methylpentane**	alkane	0.024	0.009
**n-hexane**	alkane	0.015	0.015
**methylcyclopentane**	alkane	0.018	0.000
**cyclohexane**	alkane	0.015	0.006
**2,3-dimethyl-pentane**	alkane	0.028	0.016
**3-methylhexane**	alkane	0.065	0.036
**2,2,4-trimethylpentane**	alkane	0.028	0.019
**n-heptane**	alkane	0.033	0.017
**methylcyclohexane**	alkane	0.009	0.004
**2,5-dimethylhexane**	alkane	0.020	0.009
**2,3,4-trimethylpentane**	alkane	0.018	0.009
**n-octane**	alkane	0.021	0.010
**n-nonane**	alkane	0.029	0.012
**4-methylnonane**	alkane	0.032	0.013
**n-decane**	alkane	0.028	0.011
**c-2-pentene**	alkene	0.004	0.000
**2,3,3-trimethyl-1-butene**	alkene	0.030	0.000
**1-octene**	alkene	0.011	0.000
**1-nonene**	alkene	0.010	0.000
**a-pinene**	alkene	0.006	0.000
**benzene**	aromatic	0.035	0.031
**toluene**	aromatic	0.121	0.073
**ethylbenzene**	aromatic	0.022	0.010
**m-xylene**	aromatic	0.059	0.018

Chemical	Chemical Group	Primary Pollutant Exposure (ppmC) (8:15 AM - 12:15 PM)	PCA Pollutant Exposure (ppmC) (4:30 PM - 8:30 PM)
**o-xylene**	aromatic	0.022	0.009
**n-propylbenzene**	aromatic	0.019	0.008
**m-ethyltoluene**	aromatic	0.013	0.001
**p-ethyltoluene**	aromatic	0.021	0.010
**1,2,4-trimethylbenzene**	aromatic	0.090	0.019
**sec-butylbenzene**	aromatic	0.017	0.006
**1,3-diethylbenzene**	aromatic	0.028	0.008
**1,2,3,5-tetramethylbenzene**	aromatic	0.005	0.000

Supplemental Material, Table 2: Genes identified as significantly* differentially expressed upon exposure to primary or photochemically altered (PCA) pollutants.

Dashes (-) indicate insignificant changes in expression.
* Fold change ≥ 1.5 or ≤ - 1.5, p-value < 0.05, q-value < 0.05

Gene Symbol	Primary Pollutant Fold Change (Exposed / Unexposed)	p-value	PCA Pollutant Fold Change (Exposed / Unexposed)	p-value
A1CF	-	-	-1.81	0.00276
ABCA1	-	-	-1.56	0.00494
ABCA12	-	-	-1.60	0.00261
ABCA5	-	-	-1.90	0.00149
ABCB6	-	-	-1.70	0.00001
ABCC4	-	-	-1.59	0.00002
ABCG2	-	-	-1.57	0.00124
ACAD10	-	-	-1.78	0.00075
ACAD11	-	-	-1.55	0.00078
ACSM3	-1.50	0.00606	-2.33	0.00038
ACSS2	-	-	-1.54	0.00001
ACTA2	-1.58	0.01055	-2.00	0.00239
ADD3	-	-	-1.98	0.00022
ADH1C	-	-	-1.71	0.04174
ADH6	-	-	-2.12	0.00168
ADHFE1	-	-	-1.51	0.01654
AHCYL1	-	-	-1.60	0.00109
AK3L1	-	-	-1.99	0.00053
AK7	-	-	-2.18	0.00040
AKAP12	-	-	1.67	0.00192
AKAP9	-	-	-1.73	0.00219
AKR1B1	-	-	1.55	0.00102
ALDH5A1	-	-	-1.51	0.00686
ALDH6A1	-	-	-2.50	0.00005
ALPK1	-	-	-1.82	0.00084
ALS2CR8	-	-	-1.55	0.03130
AMPD1	-	-	1.49	0.00723
ANG	-	-	-1.92	0.00069
ANKRA2	-	-	-1.82	0.00414
ANKRD1	-	-	1.68	0.01161
ANKRD18A	-	-	-1.51	0.01560
ANKRD22	-	-	2.88	0.00045
ANKRD30A	-	-	-1.87	0.00110

Gene Symbol	Primary Pollutant Fold Change (Exposed / Unexposed)	p-value	PCA Pollutant Fold Change (Exposed / Unexposed)	p-value
ANKS4B	-	-	-1.65	0.00036
ANO5	-	-	-1.52	0.00979
ANXA10	-	-	1.69	0.00491
ANXA13	-	-	-1.68	0.00414
ANXA3	-	-	1.91	0.00053
ANXA4	-	-	-1.64	0.00035
ANXA9	-	-	-1.70	0.00337
AP1S3	-	-	1.80	0.00005
APAF1	-	-	-1.52	0.00074
APH1B	-	-	-1.52	0.00342
APOBEC3C	-	-	-1.52	0.00212
APOH	-	-	-1.56	0.00202
AQP3	-	-	1.79	0.00023
AR	-	-	-1.79	0.00008
AREG	-	-	3.22	0.00010
ARFGAP2	-	-	-1.55	0.00108
ARHGAP1	-	-	-1.54	0.00038
ARID4A	-	-	-1.67	0.00244
ARID4B	-	-	-1.53	0.00671
ARID5B	-	-	-1.60	0.00007
ARL15	-	-	-1.69	0.00037
ARMCX3	-	-	-1.58	0.00332
ARRB1	-	-	-1.63	0.00000
ARSD	-	-	-1.60	0.00019
ARSE	-	-	-1.93	0.00001
AS3MT	-	-	-1.76	0.00281
ASAM	-	-	1.91	0.00731
ASPM	-	-	-1.83	0.00018
ATF6B	-	-	-1.52	0.00112
ATG2B	-	-	-1.58	0.00018
ATP8B1	-1.52	0.02013	-2.95	0.00063
ATP9A	-	-	-1.80	0.00014
AXL	-	-	1.72	0.00217
BAMBI	-	-	-1.72	0.00027
BBS9	-	-	-1.59	0.01465
BCAS3	-	-	-1.71	0.00032
BCL2L11	-	-	-2.10	0.00011
BCL2L15	-	-	-1.85	0.01002

Gene Symbol	Primary Pollutant Fold Change (Exposed / Unexposed)	p-value	PCA Pollutant Fold Change (Exposed / Unexposed)	p-value
BCMO1	**-**	**-**	**-2.49**	**0.00009**
BDH2	**-**	**-**	**-1.58**	**0.00797**
BDKRB1	**-**	**-**	**-1.57**	**0.00003**
BDKRB2	**-**	**-**	**-1.67**	**0.00396**
BLMH	**-**	**-**	**-1.51**	**0.00475**
BMPR2	**-**	**-**	**-1.82**	**0.00290**
BNIP3L	**-**	**-**	**-1.55**	**0.00010**
BTBD11	**-**	**-**	**-1.76**	**0.00003**
BTN3A1	**-**	**-**	**-1.73**	**0.00562**
BTN3A3	**-**	**-**	**-1.58**	**0.00015**
C10orf114	**-**	**-**	**1.61**	**0.00440**
C10orf57	**-**	**-**	**-1.70**	**0.00847**
C12orf27	**-**	**-**	**-1.91**	**0.00166**
C14orf106	**-**	**-**	**-1.55**	**0.02065**
C15orf51	**-**	**-**	**-1.57**	**0.01539**
C18orf58	**-**	**-**	**-1.61**	**0.00275**
C1orf63	**-**	**-**	**-1.64**	**0.00036**
C1RL	**-**	**-**	**-2.01**	**0.00000**
C1S	**-**	**-**	**-1.59**	**0.00003**
C20orf19	**-**	**-**	**-1.60**	**0.00380**
C20orf194	**-**	**-**	**-1.75**	**0.00203**
C20orf74	**-**	**-**	**-1.87**	**0.00071**
C4orf18	**-**	**-**	**-2.25**	**0.00219**
C4orf34	**-**	**-**	**-1.61**	**0.00130**
C5	**-**	**-**	**-2.07**	**0.00044**
C5orf26	**-**	**-**	**-1.50**	**0.00269**
C5orf42	**-**	**-**	**-1.64**	**0.00225**
C6orf130	**-**	**-**	**-1.54**	**0.00364**
C6orf191	**-**	**-**	**1.58**	**0.00075**
C7orf11	**-**	**-**	**1.63**	**0.00256**
C7orf68	**-**	**-**	**-1.53**	**0.00114**
C9orf3	**-**	**-**	**-2.13**	**0.00103**
CA12	**-**	**-**	**-1.52**	**0.00010**
CABYR	**-**	**-**	**-1.67**	**0.00169**
CACNA1D	**-**	**-**	**-1.87**	**0.00156**
CACNA2D1	**-**	**-**	**-1.52**	**0.00198**
CALB1	**-**	**-**	**1.62**	**0.02927**
CALCOCO1	**-**	**-**	**-1.54**	**0.00013**

Gene Symbol	Primary Pollutant Fold Change (Exposed / Unexposed)	p-value	PCA Pollutant Fold Change (Exposed / Unexposed)	p-value
CAMK2D	-	-	-1.82	0.00122
CASP4	-	-	-1.63	0.00331
CASP6	-	-	-1.60	0.00474
CCBE1	-	-	1.95	0.00131
CCBL2	-	-	-1.74	0.00035
CCDC144A	-	-	-1.55	0.00054
CCDC28A	-	-	-1.56	0.00767
CCDC34	-	-	-1.59	0.00058
CCDC80	-	-	-1.64	0.00112
CCDC99	-	-	1.60	0.00285
CCL2	1.79	0.00249	1.96	0.00143
CCNG1	-	-	-1.52	0.00018
CCNG2	-	-	-1.98	0.00375
CCPG1	-	-	-1.58	0.00182
CD177	-	-	1.95	0.03989
CD209	-	-	1.50	0.02235
CD55	-	-	1.79	0.00225
CD99L2	-	-	-1.61	0.00007
CDC14B	-	-	-1.52	0.01131
CDC25C	-	-	-1.53	0.00153
CDCA7L	-	-	-1.54	0.00104
CDCP1	-	-	1.90	0.00178
CDH1	-	-	-2.16	0.00002
CDK5RAP3	-	-	-1.67	0.00070
CDRT1	1.59	0.01225	-	-
CEACAM1	-	-	-1.84	0.00148
CEACAM5	-	-	1.52	0.00019
CENPF	-	-	-1.55	0.00048
CEP152	-	-	-1.55	0.00683
CEP70	-	-	-1.75	0.00426
CFH	-	-	-1.71	0.00089
CFHR1	-	-	-2.19	0.02652
CFHR3	-	-	-1.85	0.00017
CFI	-	-	-1.66	0.00250
CIR1	-	-	-1.55	0.00185
CIRBP	-	-	-1.88	0.00006
CLDN1	-	-	1.65	0.00159
CLMN	-	-	-1.66	0.00034

Gene Symbol	Primary Pollutant Fold Change (Exposed / Unexposed)	p-value	PCA Pollutant Fold Change (Exposed / Unexposed)	p-value
CNNM2	-	-	-1.53	0.00028
CORO2A	-	-	-1.72	0.00068
COTL1	-	-	1.51	0.00228
CPA4	-	-	1.59	0.00746
CPB2	-	-	-1.69	0.01898
CPN1	-	-	-2.02	0.00108
CRBN	-	-	-1.52	0.00023
CSGALNACT2	-	-	1.59	0.00058
CSRP1	-	-	1.70	0.00046
CST1	-	-	1.77	0.01150
CTDSP2	-	-	-1.76	0.00009
CTNND1	-	-	-1.56	0.00021
CTPS	-	-	1.80	0.00187
CTTNBP2	-	-	-1.57	0.00047
CXCL5	-	-	2.43	0.00092
CYB5A	-	-	-1.55	0.00014
CYBRD1	-	-	-1.54	0.00007
CYFIP2	-	-	-1.66	0.00007
CYHR1	-	-	-1.91	0.00028
CYP2D6	1.63	0.04999	-	-
CYP4F11	-	-	-1.66	0.00038
CYP4F12	-	-	-1.54	0.00544
CYP4F3	-	-	-1.75	0.00058
DAB2	-	-	-1.70	0.00070
DAPK1	-	-	-1.77	0.00001
DCDC2	-	-	-1.56	0.00069
DCDC5	-	-	-1.76	0.00020
DCLK1	-	-	1.90	0.00068
DEPDC4	-	-	-1.53	0.03365
DEPDC6	-	-	-2.18	0.00003
DET1	-	-	-1.58	0.00028
DGCR6	-	-	1.52	0.02151
DHCR24	-	-	-1.51	0.00004
DHFR	-	-	1.81	0.01212
DHRS3	-	-	-3.69	0.00017
DHRS9	-	-	1.56	0.02798
DHX37	-	-	1.60	0.00006
DIAPH2	-	-	-1.53	0.00949

Gene Symbol	Primary Pollutant Fold Change (Exposed / Unexposed)	p-value	PCA Pollutant Fold Change (Exposed / Unexposed)	p-value
DMXL2	-	-	-1.71	0.00070
DNAJB4	-	-	-1.55	0.01249
DND1	-	-	1.52	0.02165
DPT	-	-	1.53	0.02035
DPYD	-	-	-1.53	0.00512
DTX3L	-	-	-1.86	0.00046
DUSP1	-	-	1.65	0.00089
DUSP4	-	-	1.52	0.00138
DUSP5	-	-	1.79	0.00028
DYNC2H1	-	-	-1.63	0.00337
DZIP3	-	-	-1.55	0.01019
ECE1	-	-	-1.59	0.00002
EFHC1	-	-	-1.55	0.00639
EFNA1	-	-	-1.79	0.00405
EFNB2	-	-	1.61	0.00046
EHHADH	-	-	-1.61	0.00032
EIF2C4	-	-	-1.52	0.00280
EIF4B	-	-	-1.68	0.00510
ELF3	-	-	-1.77	0.00003
ELMO1	-	-	-1.94	0.00006
ELOVL6	-	-	-1.56	0.00533
ELP4	-	-	-1.52	0.00125
EML4	-	-	-1.52	0.00001
EMP1	-	-	1.84	0.00096
ENTPD5	-	-	-1.62	0.00211
EPB41	-	-	-1.51	0.00242
EPB41L4A	-	-	-1.80	0.00023
EPHA2	-	-	1.60	0.00280
EPHX2	-	-	-1.90	0.00134
ERAP1	-	-	-1.53	0.00020
ERBB2	-	-	-1.77	0.00002
ERBB3	-	-	-2.33	0.00023
EREG	-	-	2.83	0.00013
ESSPL	-	-	1.60	0.01585
FAM105A	-	-	-1.55	0.03236
FAM111A	-	-	-1.69	0.00133
FAM149A	-	-	-1.76	0.00333
FAM149B1	-	-	-1.52	0.00208

Gene Symbol	Primary Pollutant Fold Change (Exposed / Unexposed)	p-value	PCA Pollutant Fold Change (Exposed / Unexposed)	p-value
FAM175A	-	-	-1.55	0.00181
FAM185A	-	-	-1.56	0.04457
FAM38B	-	-	-1.88	0.00154
FAM55C	-	-	-1.55	0.00081
FAM74A3	-	-	1.53	0.00197
FARP2	-	-	-1.72	0.00120
FBXO32	-	-	-1.84	0.03748
FBXO40	-	-	1.51	0.02732
FCHSD2	-	-	-1.66	0.00440
FGA	-	-	-1.81	0.00031
FGB	-	-	-1.88	0.00010
FGFBP1	-	-	3.85	0.00009
FGFR4	-	-	-1.71	0.00013
FGG	-	-	-1.80	0.00209
FHL1	-	-	1.66	0.00166
FKBP5	-	-	-1.53	0.00162
FLI1	-	-	1.52	0.04567
FLJ11292	-	-	1.52	0.00547
FLJ35848	-	-	-1.54	0.03243
FLJ41484	-	-	-1.56	0.02264
FLJ44124	-	-	-1.55	0.00060
FLOT1	-	-	-1.65	0.00036
FMN1	-	-	-1.53	0.02740
FMO5	-	-	-2.14	0.00015
FNBP1L	-	-	-1.54	0.00003
FNIP1	-	-	-1.84	0.00347
FOXN3	-	-	-1.72	0.01025
FRAS1	-	-	-1.53	0.00459
FRK	-	-	-2.00	0.00024
FRMD3	-	-	1.69	0.00082
FSHB	-	-	1.51	0.01644
FSTL5	-	-	1.70	0.00515
FZD7	-	-	-1.82	0.00001
GAB1	-	-	-1.53	0.02435
GABARAPL1	-	-	-1.69	0.00401
GABPA	-	-	2.01	0.00008
GABRA5	-	-	1.84	0.00003
GABRE	-	-	-1.76	0.01005

Gene Symbol	Primary Pollutant Fold Change (Exposed / Unexposed)	p-value	PCA Pollutant Fold Change (Exposed / Unexposed)	p-value
GALNT12	-	-	-1.56	0.00227
GALNT4	-	-	-1.65	0.00138
GATM	-	-	-1.53	0.00008
GATS	-	-	-1.71	0.00017
GATSL1	-	-	-1.68	0.00014
GCA	-	-	-1.66	0.02017
GCOM1	1.51	0.01963	1.52	0.01866
GDF15	-	-	1.60	0.01375
GFPT2	1.60	0.00208	2.78	0.00010
GIP	-	-	-1.59	0.00122
GK	-	-	-2.16	0.00005
GLIPR1	-	-	2.20	0.00018
GLS	-	-	1.54	0.00035
GLTSCR2	-	-	-1.59	0.00000
GPAM	-	-	2.35	0.02748
GPRC5A	-	-	1.68	0.00057
GPRC5B	-	-	-2.33	0.00020
GRAMD1A	-	-	-1.59	0.00031
GREM2	-	-	1.56	0.00011
GRIP1	-	-	-1.54	0.00589
GSTA4	-	-	-1.54	0.00194
GSTM4	-	-	-1.76	0.00138
GTF2IRD2	-	-	-1.53	0.00006
GUCY1B2	-	-	-1.55	0.02367
HABP2	-	-	-1.53	0.00125
HAO1	-	-	-1.52	0.01414
HAS2	-	-	2.19	0.00276
HBEGF	-	-	1.69	0.00043
HBP1	-	-	-1.88	0.00002
HERC6	-	-	-1.68	0.00007
HFE	-	-	-1.93	0.00003
HGD	-	-	-1.86	0.00098
HIST1H1C	-	-	-1.54	0.00009
HIST1H2AB	-	-	-1.63	0.00019
HIST1H2AC	-	-	-1.57	0.00023
HIST1H2AG	-	-	-1.56	0.00076
HIST1H2AI	-	-	-1.65	0.00306
HIST1H2BM	-	-	-1.51	0.00152

Gene Symbol	Primary Pollutant Fold Change (Exposed / Unexposed)	p-value	PCA Pollutant Fold Change (Exposed / Unexposed)	p-value
HIST1H3A	-	-	-1.61	0.00256
HIST1H3E	-	-	-1.54	0.01168
HIST1H3H	-	-	-1.75	0.00148
HIST1H3J	-	-	-1.68	0.00053
HIST1H4E	-	-	-1.60	0.00275
HIST1H4H	-	-	-1.71	0.00050
HIST2H2AA3	-	-	-1.52	0.00014
HIST2H2AB	-	-	-1.62	0.00076
HIST2H2BF	-	-	-1.52	0.00093
HIST2H4A	-	-	-1.72	0.00023
HMGB2	-	-	-1.58	0.00755
HMGCL	-	-	-2.08	0.00131
HNF4A	-	-	-1.71	0.00002
HNF4G	-	-	-1.66	0.00086
HOOK1	-	-	-1.61	0.00180
HOOK3	-	-	-1.79	0.00433
HOXA2	-	-	-1.56	0.00149
HP1BP3	-	-	-1.57	0.00116
HSD17B11	-	-	-1.62	0.00054
HSD17B6	-	-	-1.50	0.04876
HSPH1	-	-	1.57	0.00072
HTR1A	-	-	1.64	0.01235
HTR3D	-	-	1.54	0.01543
ID1	-	-	-1.55	0.00006
IER3	-	-	1.56	0.01359
IFI35	-	-	-1.66	0.00049
IFIT1	-	-	-2.12	0.00579
IFT81	-	-	-1.62	0.00776
IGHA1	-	-	1.50	0.00025
IGKC	-	-	1.69	0.00480
IL11	-	-	1.85	0.01698
IL8	-	-	1.60	0.00007
INA	-	-	1.64	0.00039
INADL	-	-	-1.64	0.00020
ING4	-	-	-1.55	0.00015
IP6K2	-	-	-1.55	0.00025
IQGAP2	-	-	-1.97	0.00035
ITGA3	-	-	1.67	0.00115

Gene Symbol	Primary Pollutant Fold Change (Exposed / Unexposed)	p-value	PCA Pollutant Fold Change (Exposed / Unexposed)	p-value
ITGA6	-	-	1.61	0.00050
ITGB8	-	-	1.52	0.03105
ITGBL1	-	-	1.56	0.00139
ITPR2	-	-	-1.86	0.00137
JUN	-	-	1.69	0.00013
KCNK5	-	-	-1.53	0.00055
KCNT2	-	-	-1.75	0.00295
KDM3A	-	-	-1.50	0.00034
KIAA0922	-	-	-1.73	0.00115
KIAA1109	-	-	-1.59	0.00023
KIAA1147	-	-	-1.91	0.00001
KIAA1161	-	-	-1.66	0.00119
KIAA1199	-	-	1.66	0.00915
KIAA1370	-	-	-2.70	0.00016
KIAA1377	-	-	-1.52	0.00437
KIAA1618	-	-	-1.53	0.00043
KIAA1632	-	-	-1.55	0.00050
KIAA1712	-	-	-1.60	0.00490
KIF13B	-	-	-1.56	0.00016
KIF20A	-	-	-1.57	0.00062
KIF20B	-	-	-1.52	0.02541
KIR2DL5A	-	-	1.51	0.04814
KLHDC2	-	-	-2.08	0.00016
KLHL24	-	-	-2.44	0.00010
KRT38	-	-	1.57	0.01687
KRT80	-	-	1.68	0.00320
LAMC2	-	-	2.74	0.00002
LARGE	-	-	-1.53	0.00001
LBA1	-	-	-1.93	0.00099
LETMD1	-	-	-1.72	0.00011
LHX8	-	-	-1.68	0.00030
LIMA1	-	-	-1.70	0.00084
LIMD1	-	-	-1.51	0.00395
LITAF	-	-	-1.60	0.00000
LMO7	-	-	1.61	0.00085
LOC100130581	-	-	-1.51	0.01258
LOC100289668	-	-	-1.73	0.00711
LOC162632	-	-	-1.52	0.00058

Gene Symbol	Primary Pollutant Fold Change (Exposed / Unexposed)	p-value	PCA Pollutant Fold Change (Exposed / Unexposed)	p-value
LOC345258	-	-	1.59	0.00970
LOC440518	-	-	1.51	0.00644
LOC644714	1.72	0.03988	-	-
LOC652493	-	-	1.66	0.00876
LOC729724	-	-	1.51	0.00076
LOXL2	-	-	1.55	0.00171
LRBA	-	-	-1.53	0.00005
LRIG1	-	-	-1.53	0.00104
LRP1	-	-	-1.60	0.00103
LRRFIP1	-	-	1.75	0.00191
LXN	-	-	-3.17	0.00008
LYAR	-	-	1.52	0.01154
LYRM5	-	-	-1.64	0.01013
MAFG	-	-	-1.50	0.00030
MANBA	-	-	-1.55	0.00047
MANSC1	-	-	-1.76	0.00055
MAOA	-	-	-2.04	0.00003
MARCH4	-	-	1.61	0.00392
MARCH8	-	-	-1.57	0.00542
MARCKS	-	-	-1.64	0.00055
MATN2	-	-	-2.00	0.00034
MBOAT1	-	-	-2.24	0.00006
MCCC1	-	-	-1.66	0.00242
MCTP1	-	-	1.61	0.00115
MEIS2	-	-	-1.80	0.00026
MGAM	-	-	-1.54	0.00001
MIA2	-	-	-2.10	0.00458
MICAL2	-	-	1.54	0.00571
MIR21	-	-	-1.56	0.00251
MLEC	-	-	-1.77	0.00001
MLF1	-	-	-1.57	0.01458
MLLT4	-	-	-1.55	0.00085
MR1	-	-	-1.85	0.00105
MRAP2	-	-	-1.58	0.00189
MSI2	-	-	-1.56	0.00022
MST131	-	-	2.14	0.00035
MTMR11	-	-	-1.56	0.00059
MUT	-	-	-1.95	0.00019

Gene Symbol	Primary Pollutant Fold Change (Exposed / Unexposed)	p-value	PCA Pollutant Fold Change (Exposed / Unexposed)	p-value
MYCNOS	-	-	1.56	0.00044
MYEOV	-	-	1.62	0.00574
MYO1A	-	-	-1.66	0.00277
NAGA	-	-	-1.56	0.00399
NAP1L2	-	-	-1.57	0.00312
NAV3	-	-	2.05	0.00442
NBEA	-	-	-1.73	0.00019
NBEAL1	-	-	-1.77	0.00311
NBR1	-	-	-1.55	0.00122
NCALD	-	-	-1.51	0.00015
NCAPD2	-	-	-1.64	0.00007
NCEH1	-	-	1.52	0.00011
NCOA2	-	-	-1.51	0.00336
NDC80	-	-	-1.79	0.00277
NDRG1	-	-	-1.59	0.00516
NDRG2	-	-	-1.51	0.00030
NEB	-	-	-1.76	0.00001
NEDD4L	-	-	-1.98	0.00001
NEK11	-	-	-1.54	0.00645
NFIA	-	-	-1.95	0.00006
NFKBIA	1.51	0.00016	1.53	0.00014
NFKBIZ	-	-	-1.56	0.00523
NID2	-	-	1.77	0.00950
NIPAL3	-	-	-1.54	0.00599
NIPSNAP3A	-	-	-1.67	0.00309
NMNAT2	-	-	1.68	0.00052
NNT	-	-	-1.52	0.00002
NOSTRIN	-	-	1.62	0.00233
NOTCH2	-	-	-1.68	0.00081
NOTCH2NL	-	-	-1.57	0.00049
NPY1R	-	-	-1.74	0.00596
NR0B1	-	-	-1.52	0.00120
NR1D2	-	-	-1.53	0.00500
NR4A1	-	-	1.69	0.00307
NR5A2	-	-	-1.87	0.00024
NRG1	-	-	1.73	0.00057
NRG4	-	-	-1.81	0.00017
NRIP1	-	-	-1.66	0.00006

Gene Symbol	Primary Pollutant Fold Change (Exposed / Unexposed)	p-value	PCA Pollutant Fold Change (Exposed / Unexposed)	p-value
NRM	-	-	-1.57	0.00020
NUDT7	-	-	-1.76	0.00017
NUSAP1	-	-	-1.59	0.00367
OAS1	1.62	0.00085	2.43	0.00008
ODC1	-	-	1.66	0.00054
OPHN1	-	-	-1.96	0.00109
OR10G2	1.55	0.01301	-	-
OR2T1	-	-	1.55	0.01790
OR4A47	1.73	0.00898	1.56	0.01815
OR4C6	-	-	1.91	0.00808
OR4K1	-	-	1.53	0.03161
OR51B4	-	-	1.53	0.02610
OR51D1	1.51	0.01660	-	-
OR51L1	-	-	1.54	0.00304
OR52I2	-	-	1.50	0.04879
OR5AP2	-	-	1.58	0.03335
OR5I1	-	-	1.51	0.02120
OR9A2	-	-	1.50	0.04432
OR9Q2	-	-	1.60	0.00867
OSBPL9	-	-	-1.70	0.00006
OTUD1	-	-	-1.54	0.00205
OXTR	-	-	1.68	0.00105
P2RX4	-	-	-1.73	0.00037
P2RY4	-	-	1.54	0.01409
PAIP2B	-	-	-2.02	0.00175
PAN2	-	-	-1.61	0.00035
PAQR5	1.58	0.00319	2.19	0.00041
PAR5	-	-	-1.63	0.02688
PARP14	-	-	-1.85	0.00301
PARP9	-	-	-2.18	0.00046
PBLD	-	-	-2.57	0.00011
PCCA	-	-	-1.52	0.00462
PCDH9	-	-	-1.56	0.00041
PCMTD1	-	-	-1.66	0.00712
PCMTD2	-	-	-1.66	0.01622
PDCD4	-	-	-2.83	0.00020
PDE3A	-	-	-1.59	0.00000
PDGFC	-	-	-1.62	0.00169

Gene Symbol	Primary Pollutant Fold Change (Exposed / Unexposed)	p-value	PCA Pollutant Fold Change (Exposed / Unexposed)	p-value
PDGFRL	-	-	-1.90	0.00023
PDK2	-	-	-1.76	0.00028
PDPR	-	-	-1.52	0.00073
PDXDC2	-	-	-1.53	0.03412
PDZK1	-	-	-2.25	0.00069
PECR	-	-	-1.55	0.00431
PER2	-	-	-1.55	0.00044
PFKFB3	-	-	-1.83	0.00003
PGAP2	-	-	-1.56	0.00005
PHKB	-	-	-1.65	0.00006
PHLDA1	-	-	1.57	0.00787
PIGN	-	-	-1.57	0.00143
PIR	-	-	-1.55	0.00011
PLAU	-	-	1.75	0.00069
PLCD4	-	-	-1.53	0.03026
PLCH1	-	-	-1.99	0.00005
PLD1	-	-	-1.98	0.00015
PLEK2	-	-	1.75	0.00089
PLEKHH2	-	-	-1.64	0.00354
PMEPA1	-	-	1.51	0.00154
POF1B	-	-	-1.78	0.00037
POU1F1	-	-	1.53	0.01782
PP13439	-	-	-1.57	0.01707
PPFIBP2	-	-	-1.58	0.00008
PRKCD	-	-	-1.67	0.00020
PRKD1	-	-	-1.51	0.00039
PSG8	-	-	1.67	0.02451
PTCH2	-	-	3.64	0.00156
PTGR2	-	-	-1.55	0.00099
PTPLAD2	-	-	-1.73	0.01052
PTRF	-	-	1.65	0.00203
RAB3B	-	-	1.80	0.01271
RAP1GAP	-	-	-1.50	0.00055
RARB	-	-	-2.43	0.00014
RBKS	-	-	-1.61	0.00033
RBM14	-	-	1.51	0.00420
RBPMS	-	-	-1.51	0.00151
REXO1L1	-	-	1.50	0.00607

Gene Symbol	Primary Pollutant Fold Change (Exposed / Unexposed)	p-value	PCA Pollutant Fold Change (Exposed / Unexposed)	p-value
RFC1	**-**	**-**	**2.82**	**0.01137**
RFX5	**-**	**-**	**-1.60**	**0.00110**
RHOBTB1	**-**	**-**	**-1.72**	**0.00326**
RHOBTB3	**-**	**-**	**-2.60**	**0.00060**
RMRP	**-**	**-**	**1.51**	**0.00148**
RND1	**-**	**-**	**-1.70**	**0.00000**
RNF182	**-**	**-**	**1.80**	**0.00674**
RNF213	**-**	**-**	**-1.64**	**0.00071**
RNU11	**-**	**-**	**3.67**	**0.00002**
RNU1A	**-**	**-**	**2.73**	**0.00053**
ROBO1	**-**	**-**	**-1.63**	**0.00025**
RPPH1	**-**	**-**	**2.27**	**0.00040**
RPS27L	**-**	**-**	**-1.54**	**0.00908**
RPS6KA5	**-**	**-**	**-1.61**	**0.03867**
S100A3	**-**	**-**	**1.82**	**0.00190**
SAMD7	**-**	**-**	**1.56**	**0.01411**
SAMD9	**-**	**-**	**-1.58**	**0.00962**
SASH1	**-**	**-**	**-1.54**	**0.00060**
SCAPER	**-**	**-**	**-1.55**	**0.00306**
SCARB1	**-**	**-**	**-1.59**	**0.00036**
SCARNA17	**-**	**-**	**-1.71**	**0.00735**
SCARNA9L	**-**	**-**	**-1.52**	**0.02066**
SCD	**-**	**-**	**-1.59**	**0.00047**
SCMH1	**-**	**-**	**-1.88**	**0.00078**
SCNN1A	**-**	**-**	**-1.50**	**0.00613**
SELENBP1	**-**	**-**	**-1.64**	**0.00061**
SEMA3C	**-**	**-**	**1.51**	**0.00066**
SEMA3E	**-**	**-**	**-1.62**	**0.00373**
SEPT14	**-**	**-**	**-1.57**	**0.00871**
SERPINA6	**-**	**-**	**-1.52**	**0.00385**
SERPINB1	**-**	**-**	**-1.60**	**0.00030**
SERPINB8	**-**	**-**	**1.53**	**0.00242**
SERPINE2	**-**	**-**	**2.12**	**0.00056**
SESN3	**-**	**-**	**-2.96**	**0.00075**
SFRP1	**-**	**-**	**1.98**	**0.00127**
SFRP4	**-**	**-**	**-3.15**	**0.00197**
SFRS18	**-**	**-**	**-1.64**	**0.00018**
SH3BGRL2	**-**	**-**	**-1.51**	**0.00173**

Gene Symbol	Primary Pollutant Fold Change (Exposed / Unexposed)	p-value	PCA Pollutant Fold Change (Exposed / Unexposed)	p-value
SHMT1	**-**	**-**	**-1.62**	**0.00007**
SKAP2	**-**	**-**	**-1.68**	**0.00063**
SLC16A7	**-**	**-**	**-1.87**	**0.01007**
SLC19A3	**-**	**-**	**-1.55**	**0.00100**
SLC22A3	**-**	**-**	**-1.63**	**0.00131**
SLC23A1	**-**	**-**	**-1.77**	**0.00003**
SLC23A2	**-**	**-**	**-1.82**	**0.00014**
SLC25A27	**-**	**-**	**-1.63**	**0.00072**
SLC29A3	**-**	**-**	**-1.51**	**0.00096**
SLC2A12	**-**	**-**	**-1.62**	**0.00055**
SLC35D2	**-**	**-**	**-1.57**	**0.00050**
SLC40A1	**-**	**-**	**-1.77**	**0.00003**
SLC41A2	**-**	**-**	**-1.95**	**0.00481**
SLC44A2	**-**	**-**	**-1.64**	**0.00225**
SLC46A3	**-**	**-**	**-2.19**	**0.00010**
SLC5A3	**1.58**	**0.00021**	**1.80**	**0.00050**
SLC7A2	**-**	**-**	**-1.55**	**0.00019**
SLC9A3R1	**-**	**-**	**-1.51**	**0.00002**
SLCO4A1	**-**	**-**	**1.71**	**0.00150**
SLFN5	**-**	**-**	**-1.93**	**0.00126**
SMOX	**-**	**-**	**1.71**	**0.00078**
SMPD1	**-**	**-**	**-1.91**	**0.00009**
SNORA23	**-**	**-**	**2.06**	**0.00929**
SNORA3	**-**	**-**	**3.62**	**0.00002**
SNORA42	**-**	**-**	**6.35**	**0.00030**
SNORA52	**-**	**-**	**1.56**	**0.00372**
SNORA56	**-**	**-**	**1.77**	**0.00060**
SNORA71D	**-**	**-**	**1.60**	**0.00162**
SNORA73A	**-**	**-**	**1.92**	**0.01085**
SNORD113-3	**-**	**-**	**1.55**	**0.02204**
SNORD114-2	**-**	**-**	**1.52**	**0.00117**
SNORD115-11	**-**	**-**	**1.64**	**0.02566**
SOAT1	**-**	**-**	**-1.70**	**0.00056**
SOCS2	**-**	**-**	**1.64**	**0.00506**
SORL1	**-**	**-**	**-1.73**	**0.00003**
SPANXE	**-**	**-**	**1.54**	**0.00193**
SPATA18	**-**	**-**	**-1.57**	**0.00132**
SPATA7	**-**	**-**	**-1.54**	**0.00089**

Gene Symbol	Primary Pollutant Fold Change (Exposed / Unexposed)	p-value	PCA Pollutant Fold Change (Exposed / Unexposed)	p-value
SPG11	**-**	**-**	**-1.53**	**0.00373**
SPP1	**-**	**-**	**-1.51**	**0.00065**
SPRR2B	**-**	**-**	**1.73**	**0.04629**
SPTLC3	**-**	**-**	**-1.85**	**0.00091**
SSBP2	**-**	**-**	**-1.66**	**0.00118**
SSFA2	**-**	**-**	**1.55**	**0.00011**
ST6GAL1	**-**	**-**	**-2.01**	**0.00014**
ST8SIA4	**-**	**-**	**-1.83**	**0.00152**
STAMBPL1	**-**	**-**	**2.17**	**0.00034**
STAT4	**-**	**-**	**-2.20**	**0.00111**
STAT6	**-**	**-**	**-1.51**	**0.00003**
STC1	**-**	**-**	**1.69**	**0.00281**
STEAP2	**-**	**-**	**-1.53**	**0.00016**
STRA6	**-**	**-**	**-1.56**	**0.00983**
STX17	**-**	**-**	**-1.50**	**0.00142**
SULT2B1	**-**	**-**	**-1.69**	**0.00114**
SVEP1	**-**	**-**	**-1.85**	**0.00007**
SYCP2L	**-**	**-**	**-1.59**	**0.00681**
SYNE2	**-**	**-**	**-2.41**	**0.00008**
TAF9B	**-**	**-**	**-1.74**	**0.00036**
TAS2R5	**-**	**-**	**1.92**	**0.00015**
TBC1D5	**-**	**-**	**-1.56**	**0.00100**
TBC1D8B	**-**	**-**	**-1.63**	**0.00073**
TBCK	**-**	**-**	**-1.83**	**0.00024**
TC2N	**-**	**-**	**-1.96**	**0.00089**
TCP11L2	**-**	**-**	**-2.19**	**0.00000**
TFDP2	**-**	**-**	**-1.79**	**0.00115**
TFPI2	**-**	**-**	**1.77**	**0.00075**
TGFA	**-**	**-**	**1.53**	**0.00335**
TGFB2	**-**	**-**	**-1.67**	**0.00049**
THBD	**-**	**-**	**1.53**	**0.00012**
THG1L	**-**	**-**	**-1.56**	**0.00083**
TIGD2	**-**	**-**	**-1.85**	**0.00235**
TJP2	**-**	**-**	**-1.52**	**0.00181**
TLR1	**-**	**-**	**-2.18**	**0.00124**
TLR3	**-**	**-**	**-1.65**	**0.04429**
TM4SF20	**-**	**-**	**-2.48**	**0.00007**
TMC5	**-**	**-**	**-1.51**	**0.01022**

Gene Symbol	Primary Pollutant Fold Change (Exposed / Unexposed)	p-value	PCA Pollutant Fold Change (Exposed / Unexposed)	p-value
TMC7	-	-	-1.64	0.00134
TMEM136	-	-	-1.52	0.00435
TMEM140	-	-	-1.56	0.01972
TMEM144	-	-	-1.81	0.00091
TMEM171	-	-	1.52	0.00509
TMEM202	-	-	1.59	0.04147
TMEM37	-	-	-2.39	0.00015
TMEM50B	-	-	-1.60	0.01620
TMEM60	-	-	-1.59	0.00078
TMEM74	-	-	1.53	0.03446
TNFRSF12A	-	-	1.83	0.00138
TNFSF10	-	-	-2.26	0.00008
TNS4	-	-	2.08	0.00110
TP53	-	-	-1.59	0.00023
TP53INP1	-	-	-2.68	0.00001
TPCN1	-	-	-1.86	0.00077
TRIM31	-	-	-1.84	0.00213
TRIM52	-	-	-1.56	0.02026
TRIML2	-	-	1.60	0.01377
TSKU	-	-	-1.59	0.00001
TSPAN15	-	-	-1.56	0.00043
TST	-	-	-1.54	0.00005
TTC28	-	-	-1.68	0.00006
TTC39B	-	-	-1.63	0.01453
TTLL6	-	-	-1.53	0.00987
TUBB2C	-	-	1.51	0.01846
TXNDC16	-	-	-1.67	0.00328
TXNIP	-1.61	0.01468	-2.43	0.00160
TYR	-	-	1.73	0.03422
UBASH3B	-	-	1.82	0.00084
UGT2B15	-	-	-1.77	0.00178
UIMC1	-	-	1.93	0.00243
UNC119B	-	-	-1.58	0.00219
UNC13B	-	-	-1.59	0.00049
USP17	1.52	0.04976	1.59	0.03729
USP17L2	1.52	0.03727	1.53	0.03761
USP3	-	-	-1.50	0.00022
VCAN	-	-	-1.62	0.00012

Gene Symbol	Primary Pollutant Fold Change (Exposed / Unexposed)	p-value	PCA Pollutant Fold Change (Exposed / Unexposed)	p-value
VIL1	**-**	**-**	**-1.65**	**0.00193**
VPS13C	**-**	**-**	**-1.53**	**0.00114**
VPS39	**-**	**-**	**-1.55**	**0.00068**
VRK2	**-**	**-**	**-1.53**	**0.00264**
VWC2L	**-**	**-**	**1.57**	**0.03979**
WDR19	**-**	**-**	**-1.51**	**0.00064**
WDR69	**-**	**-**	**1.61**	**0.02320**
WEE1	**-**	**-**	**-1.93**	**0.00169**
WSB1	**-**	**-**	**-1.53**	**0.00269**
WWP1	**-**	**-**	**-1.51**	**0.00065**
XBP1	**-**	**-**	**-1.54**	**0.00334**
XCL1	**-**	**-**	**1.66**	**0.01526**
XDH	**-**	**-**	**1.52**	**0.00199**
YPEL2	**-**	**-**	**-2.04**	**0.00002**
YPEL5	**-**	**-**	**-1.93**	**0.00001**
ZBTB20	**-**	**-**	**-1.86**	**0.00095**
ZC3H6	**-**	**-**	**-1.52**	**0.01515**
ZFP14	**-**	**-**	**-1.51**	**0.01861**
ZFYVE1	**-**	**-**	**-1.54**	**0.00139**
ZKSCAN1	**-**	**-**	**-1.58**	**0.00080**
ZMYM3	**-**	**-**	**-1.55**	**0.00055**
ZNF224	**-**	**-**	**-1.53**	**0.00287**
ZNF234	**-**	**-**	**-1.52**	**0.00360**
ZNF277	**-**	**-**	**-1.54**	**0.00502**
ZNF287	**-**	**-**	**-1.65**	**0.01718**
ZNF292	**-**	**-**	**-1.62**	**0.00032**
ZNF479	**-**	**-**	**1.62**	**0.01404**
ZNF594	**-**	**-**	**-1.57**	**0.01350**
ZNF608	**-**	**-**	**-1.52**	**0.01259**
ZNF626	**-**	**-**	**1.87**	**0.03625**
ZNF654	**-**	**-**	**-1.69**	**0.01354**
ZNF704	**-**	**-**	**-1.61**	**0.00041**
ZRSR1	**-**	**-**	**1.51**	**0.00283**
ZSCAN16	**-**	**-**	**-1.52**	**0.00316**
ZSWIM6	**-**	**-**	**-1.59**	**0.00588**

Supplemental Material, Table 3: Network proteins associated with exposure to (A) primary and (B) photochemically altered (PCA) pollutant mixtures.

Network Number	Molecules in Network	p-value
**(A) Primary Pollutants Network**		
**1**	1,4-glucan,2' 5' oas,ACTA2,beta-hydroxyisovaleric acid,C13ORF15,C21ORF33, CCL2,CXCL16,CYP2D6,EDA2R,GFPT2,HNF4A,Interferon alpha,lymphotoxin-alpha1-beta2,NFkB (complex),NfkB-Nfkbia,NFKBIA,NKIRAS1,OAS1,PAQR5, PDGF BB,PIF,PNPT1,retinoic acid,RIOK3,SLC5A3,STAT4,SUMO4,TNAP, TNFAIP8,TNIP3,TRAPPC9,TXNIP,UBA7,ZFAND6	1E-25
**(B) PCA Pollutants Networks**		
**1**	A1CF,BCAS3,CDH1,Ck2,Cyp4f,CYP4F3,CYP4F12,DNAJB4,ELP4,FLJ11292,GLTSCR2,GSTA4,HGD,Histone h3,HNF4A,HNF4G,HOOK3,KIF20A,MYO1A,NMNAT2, PAQR5,PIGN,PPFIBP2,SERPINB8,SLC19A3,SLC22A3,SLC5A3,SPP1,STX17,TBCK,TMEM140,TPCN1,ZNF224,ZNF277,ZSCAN16	1E-52
**2**	ANXA3,APOH,ASPM,C14ORF106,CALCOCO1,CCDC99,CCNG1,CCNG2,CDC14B,CENPF,CYFIP2 (includes EG:26999),FAM175A,HIST1H1C,Histone H1,HMGB2, HSPH1,ING4,LETMD1,NBR1,NCAPD2,NDC80,Nuclear factor 1,NUSAP1,P2RX4, PHLDA1,PMEPA1,Ppp2c,PRKAC,ROBO1,Rsk,SCMH1,SH3BGRL2,TP53,TP53INP1,UIMC1	1E-43
**3**	ADH6,ADH1C (includes EG:126),ADHFE1,alcohol dehydrogenase,AQP3,AR, ATP9A,C7ORF68,CA12,CCDC80,CDCA7L,CIR1,CTDSP2,DHRS3,DHRS9,EFNB2, FSH,FZD7,GPRC5B,KIF20B,KLHL24,Lh,MAOA,MIR124,NDRG1,NID2,NPY1R,oxidoreductase,PTGR2,RNA polymerase II,SELENBP1,SSFA2,STAT4,TGFA,Vegf	1E-41
**4**	APH1B,BLMH,C1S,CASP4,CASP6,DTX3L,ECE1,FGFBP1,GLS,GPRC5A,HABP2,IFI35,IFIT1,IgG,IL1,Immunoglobulin,Interferon alpha,IRG,MICAL2,Mmp,NFKBIA, NGF,OAS1,PARP,PARP9,PARP14,peptidase,PIR,RPS27L (includes EG:51065), S100A3,SERPINE2,SMOX,STAT,STAT6,TNFSF10	1E-35
**5**	20s proteasome,26s Proteasome,Actin,Alpha tubulin,ARRB1,DIAPH2,DPT, DUSP1,EIF2C4,EIF4B,EPB41,ERBB2,ERBB3,GPAM,HIST2H2AB,HTR1A,ID1,INA,Insulin,LRIG1,MAP2K1/2,MARCKS (includes EG:4082),MATN2,MCCC1, NDRG2,Notch,NOTCH2,OPHN1,Pkc(s),PP2A,PTCH2,PTRF,SFRP1,TCR,XBP1	1E-35
**6**	ANKRD1,ANXA13,Cbp,CDK5RAP3 (includes EG:80279),CFHR1,CPB2,CPN1, CXCL5,DUSP5,Elastase,FGA,FGB,FGG,Fibrin,Fibrinogen,GFPT2,HAS2,IER3,IFN TYPE 1,IGKC,IP6K2,LITAF,NFkB (complex),NfkB-RelA,NFKBIZ,PPARÎ±-RXRÎ±, Pro-inflammatory Cytokine,RAP1GAP (includes EG:5909),SLC2A12,SOAT1, Stat3-Stat3,THBD,Tlr,TNFRSF12A,TXNIP	1E-32
**7**	ABCC4,AXL,BCMO1,CD209,CEACAM1,CEACAM5 (includes EG:1048),EML4, Estrogen Receptor,Ferritin,Gm-csf,Growth hormone,HFE,IL12 (complex), JAK,Ldh,LDL,LRP,MIR1,MIR21 (includes EG:406991),NEDD4L,NRG,NRG1, PDCD4,PDE3A,PI3K,PRKD1,SCNN1A,SLC9A3R1,SOCS2,ST6GAL1,STAT5a/b,TNS4,WWP1,XCL1,XDH	1E-29

Network Number	Molecules in Network	p-value
**8**	14-3-3,AHCYL1,AKAP9,ANKRA2,Calmodulin,CAMK2D,CaMKII,Creb,DET1, DGCR6,EPHX2,FOXN3,FRAS1,GABARAPL1,GABPA,GRIP1,hydrolase,ITPR2, JUN,KIF13B,LRRFIP1,MAFG,NCEH1,Nfat (family),NIPSNAP3A,PER2,PHKB,Pka ,Pka catalytic subunit,Pkg,RFC1,TUBB2C,Tubulin,UNC13B,USP3	1E-29
**9**	Adaptor protein 2,ADD3,AKAP12,Alp,Ap1,BAMBI,BMPR2,CACNA2D1,Clathrin, DAB2,EMP1,FHL1,FSHB,Hat,hCG,Histone h4,HOXA2,IFN Beta,IL8,LIMD1, Mapk,MHC Class II (complex),NBEA (includes EG:26960),NCALD,NOSTRIN, ODC1,PDGF BB,Pias,PRKCD,SCD,SFRP4,Smad2/3,STC1,Tgf beta,TGFB2	1E-27
**10**	ABCA1,C3-Cfb,CALB1,Cbp/p300,CD55,CFH,CFI,collagen,DUSP4,EHHADH, FRK,GC-GCR dimer,GDF15,HDL,ITGB8,JINK1/2,Jnk,KDM3A,LXR ligand-LXR-Retinoic acid-RXRÎ±,N-cor,NCOA2,NR0B1,NR4A1,NR5A2,NRIP1,POU1F1, RAB3B,Retinoic acid-RAR-RXR,Rxr,SCARB1,SERPINB1,T3-TR-RXR,Thyroid hormone receptor,VitaminD3-VDR-RXR,VRK2	1E-27
**11**	ACTA2,Alpha catenin,Cadherin,Calpain,CIRBP,Collagen type I,Collagen(s), CPA4,CTNND1,DAPK1,DCLK1,ERK1/2,Esr1-Esr1-estrogen-estrogen,Fgf,Fgfr, FGFR4,Focal adhesion kinase,Integrin,IntegrinÎ±,ITGA3,ITGA6,LAMC2, Laminin,Laminin1,LARGE,LMO7,LXN,MLLT4,OXTR,PDGFC,SMPD1,TFPI2, TSKU,VCAN,Vla-4	1E-25
**12**	Adaptor protein 1,ALS2CR8,AP1S3 (includes EG:130340),BRF2,CABYR, CDCA7L,CDKN2A,CES2 (includes EG:8824),CYP4F3,CYP4F11,DDX10,ELF3, FAM111A,FETUB,GABRE,HNF4A,HSD17B11,JAK1,LOXL2,MUT,PAN2,PDXDC2, PLEKHA8,retinoic acid,SERPINB8,SLC5A3,STRA6,SUZ12,TBRG1,TCF19, TMEM49,TRIM52,USP30,USP36,ZNF133	1E-23
**13**	APOL1,ATXN1,AZU1,BTN3A3,C20ORF194,CD86,CD209,CFHR3,CNN1,DPYD,D ZIP3,EIF4B,EIF4E,EIF4ENIF1,EMP1,HABP2,heparin,HIVEP1,LAMC2,LHX8,MA RCH8,MIR17 (includes EG:406952),phosphatidylinositol 3,4-diphosphate, SAA2,SERPINA6,SERPINE2,SVIL,TBC1D5,TNF,TNFRSF18,TRAF2,TRIM31,TST, XCL1,ZFYVE1	1E-23
**14**	ARSA,ARSD,ARSE,Aryl Sulfatase,CCNG1,CHST2,CLEC7A,CYBRD1,DMXL2, ELOVL6,ethanol,GREM2,HAMP,HFE2,HIST1H3A,hydrocortisone,IL2,IL13,IL13 RA2,iron,MAOA,NCAPH,norepinephrine,NPY1R,PHLDA1,PHLDA2,progestero ne,SERPINA6,SESN3,SLC40A1,SLC7A2,SUMF1,TC2N,TP53INP1	1E-20
**15**	ALDH2,ALDH5A1,ARPC2,BDKRB2,C9ORF3,CCDC28A,CCNG2,CLMN,CSRP1,EP HA2,ERCC5,FDXR,FMN1,FNBP1,HSD17B6,IER3,JAG2,KCNJ4,KIAA1199,LIMK2, LRBA,MIA2,PBLD,PIGF,PLK2,PPP1R13B,RHOC,RHOD,SHISA5,SHMT1, SLC23A2,SNRPD3,STRAP,TGFB1,TP73	1E-20
**16**	Alpha actin,AMPK,APAF1,ARID4A,BCL2L11,BNIP3L,Calcineurin protein(s), Caspase,Cdc2,CDC25C,Cyclin A,Cyclin B,Cyclin E,Cytochrome c,DHCR24, DHFR,E2f,EFHC1,FBXO32,GIP,Hdac,HIST1H2AB,HIST1H2AG,Hsp27,Hsp70, Hsp90,IQGAP2,MEF2,Mek,P38 MAPK,PFKFB3,Rb,RPS6KA5,TFDP2,WEE1	1E-20
**17**	ACTR5,ACTR8,ANK3,ARID5B,ATF7IP,BAZ1A,ERVK6,FCHSD2,GATS,HOOK1,IN O80,INO80B,INO80D,INO80E,KIAA1370,KIAA1377,KIAA1632,KIF20B,MARC H4,MGAM,MIR292 (includes EG:100049711),MIR30E (includes EG:407034), MTMR11,PEX6,PLCH1,RHOBTB1,RRBP1,RUVBL1,SCAPER,SMAD2,SMAD9,SU MO1,SVEP1,ZBTB20,ZMYND11	1E-18

Network Number	Molecules in Network	p-value
**18**	Akt,Angiotensin II receptor type 1,AREG,BDKRB1,BDKRB2,CCL2,CDCP1, CHEMOKINE,CLDN1,EGFR ligand,ERBB,ERBB4 ligand,EREG,GAB1,Gpcr, HBEGF,IFN alpha/beta,Ifn gamma,Ifnar,Ikb,IKK (complex),IL11,IL12 (family), INADL,NfkB1-RelA,NRG4,P2RY4,p70 S6k,Pik3r,Sfk,Shc,TJP2,TLR1,TLR3,Tnf	1E-18
**19**	BBS9,BTN3A1,C1R,CABP7,CORO2A,CYB561,EFCAB6,EGR2,ERAP1,GCA,GIP2,H ERC6,Hla-abc,IFI30,IFI35,IFI44,IFI47,IFNA2,IFNG,KDM5B,LARGE,MR1,MT1X, NFE2L3,OAS3 (includes EG:4940),PARP9,RNF213,SAMD9,SP110,STAMBPL1, TBC1D10A,TMEM50B,TRIM22,ZFP36,ZKSCAN1	1E-17
**20**	ABCG2,ARG1,BBS1,BCL6,beta-estradiol,BLNK,CEP152,CYB5A,CYP17A1, CYP2C9,DUSP6,EIF3A,ELF1,EPB41L2,EPB41L3,FLOT1,FMO5,GSR,HBEGF,HLA -DR,HLA-DRB1,ITGBL1,KIAA0922,MED23,MEIS2,Mhc ii (family),MIR200A (includes EG:406983),PBX1,PCDH9,RFX5,SAP30,SHBG,SSBP2,TM4SF20, TMEM37	1E-15
**21**	ARHGAP1,BCR,C5,C1q,COTL1,EFNA1,ELMO1,EPHA2,ERK,F Actin,G protein alpha,G protein alphai,G-Actin,G-protein beta,G-protein gamma,Igm,LIMA1, LRP1,p85 (pik3r),Pdgf,Pi3-kinase,PLC,PLC gamma,PLCD4,Pld,PLD1,Rac,Rap1, Ras,Ras homolog,RND1,Sapk,SORL1,SYNE2,VIL1	1E-14
**22**	ADCY10,COPS8,CST1,CTPS,DDAH2,E2F1,FNIP1,GUCY1A2,GUCY1B2,GUCY1B3, GUCY2C,GUCY2D,GUCY2E,GUCY2F,HIST1H2AC,HIST1H4H (includes EG:8365),HMGCL,HSP90AA1,KRT38,LIMA1,MLEC,MLXIP,NAGA,NFKBIL1, PECR,PNN,PRPF19,SFRS18,SLC25A3,SLC2A4,SOD2,Soluble guanylate cyclase, SRRM2,TXNL1,YWHAG	1E-13
**23**	ASF1B,BMP1,BTBD11,COL6A1,collagen,CX3CL1,dihydrotestosterone,ETV1,FG F3,GRAMD1A,HIST1H3E,HIST1H3J,Histone h4,HOXA9,KITLG,KLK2,MME, MMP2,MSI2,NEB,NNT,PARK7,PDGFRL,poly(ADP-ribose),RBM39,RDX, RPS6KB1,SFRP1,SFRS11,SLC16A7,SLC44A2,TERT,UBASH3B,UGCG,WSB1	1E-13
**24**	adenylate kinase,AK1,AK5,AK7,AK3L1,APP,ARL15,C1ORF63,C1RL,CNNM2, CRCT1 (includes EG:54544),Cytoplasmic Dynein,DYNC1LI1,DYNC2H1, FAM38B,HP,KLK6,MIR98 (includes EG:407054),MIRLET7B (includes EG:406884),NAV3,prostaglandin E2,PROZ,PRSS1 (includes EG:5644),PRSS2 (includes EG:25052),PRSS3 (includes EG:5646),PTN,PTPLAD2,REG1A, SERPINE2,SLC31A1,TCOF1,Trypsin,YPEL5,ZNF654	1E-13
**25**	AHSG,ALB,AMBP,APOC3,ATG2B,CDC45L,CDKN1B,CLCA2 (includes EG:9635), COPS3,CYHR1,HAO1,HNF1A,HPX,LGALS3,MIR26A1,MIR291B,MLF1,NFIA, NFIB,NFIC,NFIX,NR1D1,NR1D2,OSBPL9,PLEK2,PRSS1 (includes EG:5644), PRSS3 (includes EG:5646),SERPING1,SLC25A27,TBC1D8B,Tcf 1/3/4,TCF7L2 (includes EG:6934),TMOD2,ZNF292,ZNF608	1E-12

Supplemental Material, Table 4: Disease categories identified by DAVID analysis as enriched in cells exposed to (A) primary pollutants, and (B) PCA pollutants.

DAVID's functional annotation tool was used to identify associated diseases (Dennis 2003, Huang 2009a, Huang 2009b). P-values are calculated using a modified Fisher's Exact test (as described in Hosack 2003). Overlapping disease categories identified through the Ingenuity Systems® analysis are also detailed.

DAVID's Enriched Disease Category	p-value	Genes	Ingenuity System's Related Enriched Disease Category
**(A) Primary Pollutant Exposure**			
**diabetes, type 1**	0.008	*CCL2, CYP2D6, NFKBIA, OAS1*	
**multiple sclerosis**	0.050	*CCL2, CYP2D6, OAS1*	
**H. pylori infection**	0.067	*CCL2, CYP2D6*	
**colorectal cancer**	0.074	*CCL2, CYP2D6, NFKBIA*	Cancer
**liver cancer**	0.100	*CYP2D6, NFKBIA*	Cancer
**(B) PCA Pollutant Exposure**			
**myocardial infarct; thromboembolism, venous; thrombosis, deep vein**	0.006	*FGG, FGA, FGB*	Cardiovascular Disease
**fibrinogen**	0.006	*FGG, FGA, FGB*	
**angina**	0.012	*FGA, FGB, ITGA3, CPB2*	Cardiovascular Disease
**thrombosis, deep vein**	0.017	*FGG, THBD, FGA, FGB, CPB2*	
**fibrinogen myocardial infarct**	0.020	*FGG, FGA, FGB*	Cardiovascular Disease
**fibrinogen heart disease, ischemic tissue plasminogen activator level**	0.020	*FGG, FGA, FGB*	Cardiovascular Disease
**iron levels**	0.029	*CYBRD1, HFE, SLC40A1*	
**fetal loss, late**	0.035	*THBD, FGB, HFE, ITGA3*	Embryonic Development
**thromboembolism, venous**	0.040	*LRP1, THBD, FGA, FGB, CPB2, HABP2*	
**myocardial infarction**	0.054	*FGG, THBD, FGA, FGB, HFE, BDKRB2, CPB2*	Cardiovascular Disease
**atherosclerosis, coronary**	0.054	*CCL2, ADH1C, EPHX2, ITGA3, BDKRB1, BDKRB2, ABCA1, LRP1, ECE1, THBD, FGB, CFH, SCARB1*	
**colorectal cancer**	0.081	*ODC1, SHMT1, CCL2, GSTA4, IL8, CXCL5, TP53, ADH1C, NFKBIA, HFE, CDH1, DHFR, DPYD, GDF15, PLAU*	Cancer
**migraine**	0.091	*EFHC1, AR, MAOA, GABRA5, SLC25A27*	
**atherosclerosis, coronary atherosclerosis, generalized**	0.091	*FGG, FGA*	
**post-trauma fibrinogen increase**	0.091	*FGA, FGB*	
**haemochromatosis**	0.091	*HFE, SLC40A1*	
**Aalpha and gamma fibrinogen plasma levels**	0.091	*FGG, FGA*	

Supplemental Material, Table 5: Transcription factors predicted to regulate genes modified upon exposure to (A) both primary and PCA pollutants, (B) primary pollutants, and (C) PCA pollutant mixtures.

Transcription Factor	TRANSFAC Accession Number	Predicted Gene Targets (Targets are Genes Identified as Differentially Expressed)	Targets' Expression Direction	p-value
**(A) Overlapping Transcription Factors (Primary and PCA Pollutants)**				
**HNF-1**	M00132	See tables (B) and (C) below	Decreased	0.003 (average)
**NF-Y**	M00287	See tables (B) and (C) below	Decreased	0.005 (average)
**Oct-1**	M00137	See tables (B) and (C) below	Decreased	0.014 (average)
**GATA-1**	M00127	See tables (B) and (C) below	Decreased	0.017 (average)
**FOXO4**	M00472	See tables (B) and (C) below	Decreased	0.022 (average)
**Evi-1**	M00078	See tables (B) and (C) below	Decreased	0.039 (average)
**(B) Primary Pollutant Mixture**				
**PPARalpha**	M00242	*ACSM3, ACTA2, TXNIP*	Decreased	0.001
**PAX**	M00808	*CYP2D6, OAS1, PAQR5, SLC5A3*	Increased	0.002
**HNF-1**	M00132	*ACSM3, TXNIP*	Decreased	0.006
**UF1H3BETA**	M01068	*CYP2D6, GFPT2, NFKBIA, PAQR5, SLC5A3*	Increased	0.006
**TBX5**	M01020	*NFKBIA, PAQR5, SLC5A3*	Increased	0.006
**SRF**	M00186	*ACTA2*	Decreased	0.007
**NF-Y**	M00287	*ACSM3, TXNIP*	Decreased	0.009
**GATA-1**	M00127	*ACSM3, ACTA2*	Decreased	0.015
**MZF1**	M00083	*CYP2D6, SLC5A3*	Increased	0.017
**Oct-1**	M00137	*ACSM3, TXNIP*	Decreased	0.027
**Hmx3**	M00433	*ACTA2, TXNIP*	Decreased	0.029
**RREB-1**	M00257	*CYP2D6, GFPT2, NFKBIA, OAS1*	Increased	0.029
**Evi-1**	M00078	*TXNIP*	Decreased	0.035
**HNF-4**	M00134	*CYP2D6, OAS1, SLC5A3*	Increased	0.036
**COUPTF**	M01036	*CYP2D6, GFPT2, SLC5A3*	Increased	0.041
**FOXO4**	M00472	*ACTA2, TXNIP*	Decreased	0.043
**Freac-3**	M00291	*TXNIP*	Decreased	0.045
**(C) PCA Pollutant Mixture**				
**FOXO4**	M00472	*A1CF, ACTA2, ALPK1, APAF1, APH1B, ARSE, ASPM, BCL2L11, BCMO1, BDH2, BNIP3L, C4orf18, CCDC28A, CCDC34, CCDC80, CCPG1, CDC25C, CDCA7L, CENPF, CFHR3, CFI, CIR1, CORO2A, CRBN, CTDSP2, CTNND1, CTTNBP2, DEPDC4, DIAPH2, EFNA1, EIF2C4, ELOVL6,*	Decreased	9.45E-09

Transcription Factor	TRANSFAC Accession Number	Predicted Gene Targets (Targets are Genes Identified as Differentially Expressed)	Targets' Expression Direction	p-value
		EPHX2, FBXO32, FGB, FRK, GATM, HSD17B11, ID1, IFT81, IQGAP2, KIAA1370, KIF20A, MANSC1, MARCKS, MCCC1, MGAM, MUT, NAP1L2, NCOA2, NDRG1, NR0B1, NRG4, OSBPL9, PAIP2B, PBLD, PCMTD1, PDCD4, PDE3A, PDZK1, POF1B, RBKS, RND1, RPS6KA5, SESN3, SKAP2, SLC2A12, SLC35D2, SPTLC3, ST8SIA4, SYCP2L, TFDP2, TGFB2, TLR3, TMEM140, TMEM37, TNFSF10, TXNDC16, TXNIP, VCAN, VRK2, WEE1, WWP1, ZBTB20, ZNF608, ZNF654		
**HNF-1**	M00132	*A1CF, ABCA12, ABCG2, ACSM3, ADH6, ALPK1, ANG, ANKRA2, ANKS4B, ANXA13, APOH, ARSE, C4orf18, C5, C7orf68, CASP4, CEACAM1, CEP152, CFHR3, CFI, CPB2, CYB5A, DAB2, ELMO1, FAM38B, FGA, FGB, FGG, FRK, GLTSCR2, HABP2, HAO1, HIST2H2BA, HNF4A, HOOK3, IP6K2, MANSC1, MARCKS, MGAM, MIA2, MTMR11, NAP1L2, NEB, NIPAL3, NIPSNAP3A, NNT, NR5A2, NRM, OPHN1, PLCH1, RPS6KA5, SAMD9, SEMA3E, SERPINA6, SLC25A27, SLC41A2, SLC7A2, SPATA7, STEAP2, TBC1D5, THG1L, TLR1, TLR3, TM4SF20, TMEM136, TMEM144, TMEM37, TXNIP, VPS13C, VRK2, WSB1, YPEL2, ZNF654, ZNF704*	Decreased	6.23E-06
**TEF**	M00672	*ABCA12, ADHFE1, AKAP9, AR, ARID5B, ASPM, BCAS3, BTBD11, BTN3A1, C1RL, C1S, C4orf34, C5, C5orf42, CCDC28A, CCNG2, CCPG1, CENPF, CFH, CFHR1, CFHR3, CRBN, DAB2, DMXL2, DNAJB4, DZIP3, ELOVL6, FAM149A, FAM38B, FGB, FLOT1, GRIP1, GSTM4, HIST2H2AA3, HIST2H4A, HSD17B6, ING4, KIAA0922, KIAA1377, KIF13B, MANSC1, MIA2, NAP1L2, NEB, NFKBIZ, NIPAL3, NIPSNAP3A, NR0B1, NRG4, NRM, P2RX4, PBLD, PLCD4, RFX5, SEMA3E, SESN3, SFRS18, SLC16A7, SPTLC3, SYCP2L, TBC1D8B, TIGD2, TLR1, TLR3, TRIM31, TSKU, TTC28, UGT2B15, UNC119B, VPS13C, ZC3H6, ZNF292, ZNF594, ZNF654, ZSCAN16*	Decreased	1.18E-05
**NF-Y**	M00287	*ABCA5, ABCB6, ACSM3, ADHFE1, ALDH6A1, ARFGAP2, ARHGAP1, ARID4A, ARID4B, ASPM, ATF6B, BAMBI, BLMH, BTBD11, BTN3A1, C4orf18, C6orf130, CABYR, CCBL2, CCDC28A, CCDC34, CCNG1, CCNG2, CDC14B, CDH1, CDK5RAP3, CENPF, CEP152, CFI, CIRBP, CNNM2, CPB2, CTDSP2, CYFIP2, CYP4F12, DAPK1, DCDC2, DHCR24, ECE1, EHHADH, ELP4, FAM105A, FAM55C, FARP2, FNIP1, GCA, GK, HBP1, HFE, HIST1H2AB, HIST1H2AC, HIST2H2AA3, HIST2H2BA, HMGB2, HOOK1, HP1BP3, ID1, ITPR2, KCNT2, KDM3A, KIAA1377, KIF20A, KIF20B, KLHL24, LARGE, LETMD1, LRBA, LRIG1, MAOA, MARCKS, MBOAT1, MLEC, NCALD, NCAPD2, NDRG2, NEB, NEDD4L, NRIP1, NUSAP1, PAIP2B, PAN2, PCDH9, PDCD4, PDK2, PDZK1, PER2, PGAP2, PIGN, PLEKHH2, RFX5, RND1, RPS6KA5, SAMD9, SCD, SERPINA6, SESN3, SHMT1, SKAP2, SLC25A27, SLC46A3, SLC9A3R1, ST8SIA4, TFDP2, TMEM50B, TP53, TPCN1, TSPAN15, TXNDC16, TXNIP, WEE1, XBP1,*	Decreased	2.31E-05

Transcription Factor	TRANSFAC Accession Number	Predicted Gene Targets (Targets are Genes Identified as Differentially Expressed)	Targets' Expression Direction	p-value
		YPEL2, YPEL5, ZBTB20, ZNF287, ZNF594, ZSCAN16		
**LEF1**	M00805	*A1CF, ABCA1, ABCA12, ABCA5, ACAD10, ACAD11, ACSS2, ACTA2, ADD3, ADH1C, ALDH5A1, ANG, ANXA9, APOBEC3C, APOH, AR, ARHGAP1, ARMCX3, ARSD, ARSE, BCAS3, BCL2L11, BDH2, BDKRB1, BDKRB2, BLMH, BTN3A1, BTN3A3, C14orf106, C20orf74, C5, C6orf130, C9orf3, CABYR, CCDC28A, CCDC34, CCDC80, CCNG1, CCPG1, CD99L2, CDC25C, CDK5RAP3, CEACAM1, CFHR1, CFHR3, CIR1, CNNM2, CPB2, CPN1, CTNND1, CTTNBP2, CYBRD1, CYP4F11, DAB2, DEPDC4, DET1, DHRS3, DIAPH2, DMXL2, DNAJB4, EFHC1, EFNA1, ELF3, ELMO1, ELOVL6, ELP4, EPHX2, ERAP1, ERBB2, FAM111A, FAM149A, FAM55C, FGA, FGB, FKBP5, FNIP1, FRK, FZD7, GABARAPL1, GATSL1, GCA, GK, GLTSCR2, HABP2, HAO1, HIST1H2AB, HIST1H2AG, HIST2H2AA3, HOXA2, HSD17B11, ID1, KCNT2, KDM3A, KIAA0922, KIAA1377, KIF13B, KIF20A, KLHDC2, KLHL24, LETMD1, LRBA, LRIG1, LRP1, LXN, LYRM5, MANBA, MANSC1, MARCKS, MATN2, MCCC1, MIA2, MLEC, MR1, MRAP2, MSI2, MTMR11, NAP1L2, NCALD, NEB, NEDD4L, NFIA, NIPAL3, NIPSNAP3A, NOTCH2, NOTCH2NL, NPY1R, NR1D2, NR5A2, NRIP1, NUDT7, NUSAP1, PAIP2B, PAN2, PARP14, PARP9, PBLD, PDCD4, PDE3A, PDGFC, PDGFRL, PDK2, PFKFB3, PLCD4, PLCH1, POF1B, PRKCD, RAP1GAP, RARB, RBKS, RHOBTB1, RNF213, SAMD9, SCAPER, SELENBP1, SERPINB1, SESN3, SFRS18, SH3BGRL2, SLC16A7, SLC19A3, SLC23A2, SLC25A27, SLC29A3, SLC2A12, SLC40A1, SLC41A2, SLC44A2, SPATA7, ST8SIA4, STEAP2, STX17, SVEP1, SYCP2L, SYNE2, TBC1D8B, TBCK, TC2N, TFDP2, TGFB2, TLR3, TM4SF20, TMEM136, TMEM37, TNFSF10, TRIM31, TTC39B, TTLL6, USP3, VCAN, VPS13C, VRK2, WEE1, WSB1, YPEL5, ZFP14, ZKSCAN1, ZNF224, ZNF287, ZNF594, ZNF608, ZNF654, ZNF704, ZSCAN16*	Decreased	4.37E-04
**PLZF**	M01075	*A1CF, ACAD11, ACSM3, ADHFE1, ANO5, APH1B, AR, ASPM, BCAS3, BCL2L15, C1S, C4orf18, C4orf34, C5orf42, CCDC34, CDK5RAP3, CENPF, CFH, CFI, CLMN, CPB2, CRBN, CYB5A, DAB2, DAPK1, ELP4, FAM149B1, GABARAPL1, GCA, GSTA4, HAO1, HIST2H4A, HMGB2, HOOK3, IFIT1, KDM3A, KIAA0922, KIAA1370, KIAA1632, KIF13B, KLHDC2, KLHL24, LYRM5, MCCC1, MGAM, MIA2, NBEAL1, NCALD, NEB, NFIA, NFKBIZ, NIPSNAP3A, NUDT7, NUSAP1, PAIP2B, PBLD, PCMTD1, RHOBTB3, SEMA3E, SESN3, SH3BGRL2, SLC16A7, SLC41A2, SSBP2, TBC1D5, TIGD2, TM4SF20, TMEM136, ZNF292, ZNF608, ZNF654*	Decreased	4.58E-04
**Oct-1**	M00161	*ABCB6, ACSM3, ADH6, ADHFE1, ANXA13, ARFGAP2, ASPM, C4orf18, CASP4, CCDC34, CEACAM1, CEP70, CFHR1, CORO2A, CRBN, DAB2, DNAJB4, ELF3, ENTPD5, FAM105A, FGFR4, FOXN3, GABARAPL1, GATM, HABP2, HIST1H2AC, HIST2H2AA3, HIST2H2BA, KCNT2,*	Decreased	6.30E-04

Transcription Factor	TRANSFAC Accession Number	Predicted Gene Targets (Targets are Genes Identified as Differentially Expressed)	Targets' Expression Direction	p-value
		KIAA1109, LHX8, LYRM5, MARCKS, MBOAT1, MGAM, MTMR11, NBEAL1, NEB, OSBPL9, PARP9, PDZK1, PLCD4, POF1B, RHOBTB1, RHOBTB3, SCD, SCNN1A, SEMA3E, SLC19A3, STAT4, TBC1D5, TBC1D8B, TLR1, TSPAN15, WSB1, WWP1, YPEL5, ZNF292		
**HOXA4**	M00640	*ANKRD1, CALB1, CCBE1, CLDN1, CXCL5, EREG, FBXO40, ITGA3, ITGBL1, LMO7, SERPINB8*	Increased	8.03E-04
**HNF-1**	M00206	*A1CF, ABCA12, ADH6, ALPK1, ANG, ANKS4B, ANXA13, BAMBI, BDKRB1, C4orf18, C5orf42, CALCOCO1, CCNG2, CTNND1, DAB2, DIAPH2, DYNC2H1, FGA, FRK, HIST2H4A, HP1BP3, HSD17B11, ING4, IQGAP2, LXN, LYRM5, MIA2, NAP1L2, NEB, NIPAL3, NNT, NRM, PCCA, PLCH1, RARB, SAMD9, SLC19A3, SLC23A1, SLC2A12, SLC35D2, SPATA18, SPTLC3, STRA6, THG1L, TLR3, TM4SF20, TMEM37, UGT2B15, VPS13C, YPEL2, ZNF654*	Decreased	0.001
**CDX**	M00991	*A1CF, ABCA5, ACAD11, ACSM3, ALDH6A1, ANG, ARID4B, ARID5B, ARMCX3, ASPM, BBS9, BCMO1, BDH2, BTN3A3, C5orf42, CCDC28A, CENPF, CFH, CFHR1, CFHR3, CRBN, DEPDC6, DIAPH2, DNAJB4, DPYD, DYNC2H1, ENTPD5, FAM149B1, FAM175A, FGB, HBP1, HFE, HIST1H2AC, HIST2H4A, HNF4G, HOOK3, HOXA2, KCNK5, LYRM5, MARCKS, MCCC1, MIA2, NAP1L2, NBEAL1, NCALD, NRG4, PCMTD1, POF1B, SESN3, SHMT1, SKAP2, SLC16A7, SLC19A3, SLC41A2, SPATA18, SPP1, ST8SIA4, TBC1D5, TLR1, TLR3, TMEM136, TXNIP, UGT2B15, WWP1, XBP1, ZBTB20, ZC3H6, ZNF224, ZNF287, ZNF292, ZNF608, ZNF654, ZSCAN16*	Decreased	0.001
**SRY**	M00148	*A1CF, ABCA1, ABCG2, ADH1C, ANG, ANKRA2, ANXA13, APH1B, APOH, ARFGAP2, ARID5B, ARSD, ARSE, AS3MT, ASPM, BAMBI, BBS9, BCMO1, BDH2, BLMH, BMPR2, BNIP3L, BTN3A1, BTN3A3, C1orf63, C1S, C5, C7orf68, CCDC28A, CCNG2, CDC14B, CDC25C, CDCA7L, CEP152, CFHR3, CIR1, CORO2A, CRBN, CTTNBP2, DNAJB4, EFHC1, EFNA1, EIF4B, ELMO1, ELOVL6, EPHX2, ERAP1, FAM111A, FAM149B1, FAM38B, FBXO32, FGB, FGG, FOXN3, FRK, FZD7, GABARAPL1, GATM, GATSL1, GK, HABP2, HBP1, HFE, HIST1H2AC, HIST2H4A, HP1BP3, IFIT1, IFT81, INADL, ING4, IP6K2, IQGAP2, KIAA1632, LITAF, LYRM5, MARCKS, MCCC1, MGAM, MR1, NAP1L2, NCALD, NDRG1, NFIA, NPY1R, NRG4, NUDT7, PAIP2B, PBLD, PCMTD1, PDCD4, PPFIBP2, PTPLAD2, RFX5, RND1, RNF213, SAMD9, SASH1, SERPINA6, SFRP4, SFRS18, SH3BGRL2, SKAP2, SLC16A7, SLC2A12, SLC35D2, SLC7A2, SMPD1, SPATA18, SPATA7, SPP1, SPTLC3, STAT6, STX17, SYCP2L, SYNE2, TBC1D8B, TFDP2, THG1L, TLR1, TLR3, TM4SF20, TMEM136, TMEM144, TMEM37, TMEM50B, TPCN1, TRIM31, TST, TXNIP, UNC13B, VPS13C, VRK2, ZBTB20, ZNF287, ZNF704*	Decreased	0.002
**Bach2**	M00490	*CCDC99, CEACAM5, COTL1, CST1, DCLK1, FHL1, FLI1, IL11, IL8, KRT80, LAMC2, NID2, NR4A1, OR4C6, PSG8,*	Increased	0.003

Transcription Factor	TRANSFAC Accession Number	Predicted Gene Targets (Targets are Genes Identified as Differentially Expressed)	Targets' Expression Direction	p-value
		RAB3B, RNF182, SEMA3C, SERPINB8, SNRPN, TFPI2, TMEM171		
**S8**	M00099	*A1CF, ACAD10, ADH1C, ANKS4B, ASPM, BAMBI, C1S, C5, C5orf42, CACNA1D, CALCOCO1, CDCA7L, CENPF, CYFIP2, DAB2, DCDC2, DIAPH2, DMXL2, DNAJB4, ELMO1, ELP4, FAM55C, FNIP1, HAO1, HERC6, HFE, HIST1H2AC, IFIT1, KCNT2, KIF13B, LYRM5, MARCKS, MGAM, NAP1L2, NBEAL1, NCALD, NCAPD2, NIPSNAP3A, NRG4, PDZK1, PECR, PIR, PLCH1, RHOBTB3, SAMD9, SASH1, SEMA3E, SH3BGRL2, SLC16A7, TIGD2, TLR1, TMEM60, TTLL6, YPEL2, ZC3H6, ZFYVE1, ZNF704*	Decreased	0.003
**C/EBPgamma**	M00622	*ADH1C, ADH6, ALPK1, ANXA13, APOH, ARSD, BCMO1, C9orf3, CABYR, CACNA1D, CALCOCO1, CASP4, CCDC28A, CDC14B, CEP70, CFHR1, CRBN, CYBRD1, DAB2, DCDC2, DMXL2, DZIP3, FAM111A, FGB, IQGAP2, KIAA0922, KIAA1377, KIF13B, LHX8, MCCC1, MEIS2, NEK11, NRG4, PAN2, PBLD, PCMTD2, PDCD4, PECR, PLCH1, PLEKHH2, PTGR2, RPS6KA5, SEMA3E, SLC2A12, SLC41A2, SPG11, SPP1, STAT4, SYCP2L, TMEM140, UGT2B15, VPS13C, ZNF292*	Decreased	0.003
**Nkx6-2**	M00489	*ADH1C, ADH6, ANG, ANKS4B, ASPM, BBS9, C5, C5orf42, CALCOCO1, CCDC34, CEACAM1, CEP152, CYB5A, DMXL2, ELMO1, ENTPD5, EPHX2, FAM38B, FAM55C, FGB, FGG, FLOT1, FNIP1, FRK, HFE, HIST2H4A, HNF4G, KIAA1109, KIAA1370, KIAA1712, LHX8, LYRM5, MANSC1, MARCKS, MCCC1, MIA2, NAP1L2, NFIA, NIPAL3, NIPSNAP3A, NNT, NR0B1, PAN2, PDCD4, PLCH1, RPS6KA5, SCAPER, SESN3, SH3BGRL2, SHMT1, SORL1, TBC1D5, TMEM136, TNFSF10, TXNIP, UGT2B15, ZC3H6, ZKSCAN1, ZNF292*	Decreased	0.005
**FOXJ2**	M00423	*A1CF, ABCG2, ADH1C, ANXA13, ARID5B, ARMCX3, ASPM, BLMH, BTN3A3, C4orf18, C4orf34, C5orf42, CCDC28A, CCDC80, CFHR3, CFI, CPB2, DPYD, DTX3L, DYNC2H1, DZIP3, FGB, FGG, FNIP1, HSD17B11, KIAA1370, KIF20A, MANSC1, MARCKS, MGAM, MUT, NAP1L2, NBEAL1, NFIA, NFKBIZ, NIPSNAP3A, NRG4, NUDT7, PBLD, PCCA, POF1B, SHMT1, SKAP2, SLC16A7, SORL1, SPP1, SYCP2L, TBC1D5, TM4SF20, YPEL5, ZMYM3, ZNF292, ZNF654*	Decreased	0.005
**C/EBPalpha**	M00116	*ADH1C, ADHFE1, ALPK1, ARMCX3, C9orf3, CCNG2, CFH, CFHR1, CPB2, DEPDC4, DHRS3, DMXL2, ELP4, ENTPD5, FAM38B, FGA, GCA, HIST1H2AB, HNF4G, INADL, ING4, KIAA0922, LHX8, LRBA, LXN, MANBA, MANSC1, MR1, MUT, NBEAL1, NDRG2, NEK11, PBLD, PCMTD2, PFKFB3, SLC7A2, ST8SIA4, STEAP2, TIGD2, TLR3, TSKU, ZC3H6, ZNF292, ZNF608, ZNF654, ZSCAN16*	Decreased	0.006
**STAT5A**	M00499	*ANKRD1, ANXA10, C6orf191, CCL2, CLDN1, CPA4, FSTL5, GABRA5, GPRC5A, HBEGF, ITGA3, ITGBL1, KRT38, OR4C6, OR51B4, OXTR, SAMD7, SERPINE2, SLC5A3, SPRR2B, TFPI2, WDR69*	Increased	0.007
**NF-AT**	M00302	*ABCA1, ACSS2, ADHFE1, ALS2CR8, BTN3A1, C20orf74, C5orf42, CCBL2, CCDC80, CEP152, CFHR3, CYB5A,*	Decreased	0.009

Transcription Factor	TRANSFAC Accession Number	Predicted Gene Targets (Targets are Genes Identified as Differentially Expressed)	Targets' Expression Direction	p-value
		DIAPH2, EIF2C4, ELOVL6, ELP4, FAM105A, FAM55C, FGFR4, HOXA2, HP1BP3, LIMA1, LRBA, MARCKS, NFKBIZ, NR5A2, PAIP2B, PER2, SFRP4, SLC25A27, SLC29A3, STAT4, STX17, SYNE2, TBC1D5, TGFB2, TLR1, TM4SF20, TMEM144, TNFSF10, TP53INP1, TTC39B, UGT2B15, ZNF287, ZNF654		
**NF-AT**	M00935	*ABCA5, ARID4B, ARRB1, C4orf18, CEP152, CEP70, CYP4F3, DMXL2, FBXO32, HFE, HIST2H2BA, KIAA1377, LRBA, MARCKS, NIPSNAP3A, NNT, NUSAP1, OPHN1, PBLD, PDGFRL, RARB, SEMA3E, SLC16A7, SYNE2, TBC1D5, TCP11L2, TMEM144, TP53, TTC39B, YPEL2, ZNF654*	Decreased	0.009
**Freac-4**	M00292	*AMPD1, ANKRD1, ANXA10, CCL2, CD55, DHRS9, DUSP4, EMP1, GDF15, HAS2, LMO7, NAV3, NMNAT2, OXTR, PHLDA1, RNF182, SNRPN, SPRR2B*	Increased	0.010
**TFIIA**	M00707	*AKAP12, ASAM, AXL, CLDN1, CXCL5, DGCR6, DHX37, ITGA3, KIAA1199, ODC1, PMEPA1, STAMBPL1, TGFA, XCL1*	Increased	0.011
**PLZF**	M01075	*AKAP12, ANKRD22, ANXA10, CCBE1, CPA4, DHX37, DUSP1, EMP1, FBXO40, FSHB, GPRC5A, HTR3D, IL11, IL8, ITGBL1, KRT38, OR4C6, OR51B4, OXTR, POU1F1, SAMD7, SOCS2*	Increased	0.013
**HFH-1**	M00129	*A1CF, ACTA2, ADHFE1, APH1B, APOH, ARID4B, ARID5B, BDH2, BDKRB2, C14orf106, C4orf18, CASP4, CCDC28A, CLMN, CRBN, DAB2, DEPDC6, DMXL2, DNAJB4, DYNC2H1, ENTPD5, ERAP1, FAM105A, FAM175A, FGB, FGG, FNIP1, HAO1, HIST1H2AC, HIST2H2AA3, HSD17B11, IFT81, ITPR2, LITAF, LXN, MGAM, MSI2, NAP1L2, NCOA2, NIPSNAP3A, NR5A2, NRG4, NUDT7, PARP9, PBLD, PCMTD1, RHOBTB1, RHOBTB3, SASH1, SEMA3E, SKAP2, SLC16A7, SLC19A3, SLC2A12, SLC7A2, SORL1, SPTLC3, SYCP2L, SYNE2, TBC1D8B, TM4SF20, TMEM136, TMEM140, UNC119B, VRK2, WWP1, ZMYM3, ZNF654*	Decreased	0.013
**TBP**	M00471	*AMPD1, ANKRD22, ANXA10, CALB1, CCBE1, CPA4, DHRS9, DPT, FBXO40, FLI1, ITGBL1, NR4A1, OR4C6, OR51B4, POU1F1, SPRR2B, SSFA2, STAMBPL1, TFPI2*	Increased	0.014
**ZID**	M00085	*AP1S3, CDCP1, CEACAM5, CPZ, CTPS, DND1, FSTL5, GFPT2, ITGB8, ITGBL1, LRRFIP1, LYAR, MICAL2, NMNAT2, PTRF, RAB3B, SLCO4A1, SOCS2, TMEM171, TMEM74, XDH*	Increased	0.014
**HMGIY**	M01010	*ABCG2, ACTA2, ANKS4B, ANXA4, ARFGAP2, BCL2L11, BDKRB1, BTN3A1, CCBL2, CCNG1, CFH, CPB2, DHRS3, DYNC2H1, ELOVL6, ELP4, FGB, HIST2H2BA, IQGAP2, KCNT2, KDM3A, KIAA0922, KIAA1377, KIF20A, LHX8, LYRM5, MCCC1, NFIA, NPY1R, NR5A2, NUDT7, PAIP2B, PLCH1, SAMD9, SFRS18, SLC35D2, SLC41A2, SYCP2L, TBC1D8B, TIGD2, TLR3, TM4SF20, TPCN1, WEE1, XBP1, ZNF608*	Decreased	0.014
**TATA**	M00252	*AMPD1, DPT, FGFBP1, GDF15, GLIPR1, IL8, JUN,*	Increased	0.015

Transcription Factor	TRANSFAC Accession Number	Predicted Gene Targets (Targets are Genes Identified as Differentially Expressed)	Targets' Expression Direction	p-value
		KIAA1199, NR4A1, SERPINB8, SNRPN, STAMBPL1, STC1		
**SOX**	M01014	*C6orf191, CALB1, CCBE1, DUSP4, FGFBP1, FLI1, FRMD3, ITGB8, ITGBL1, NAV3, NMNAT2, NRG1, OAS1, SOCS2, TNFRSF12A*	Increased	0.015
**NF-E2**	M00037	*ANKRD22, AQP3, CCDC99, COTL1, CPZ, DGCR6, FGFBP1, GLIPR1, HAS2, HTR3D, IL11, KIAA1199, KRT80, NAV3, OR51B4, RAB3B, SERPINB8, TNS4*	Increased	0.017
**DEC**	M00997	*AKAP12, ASAM, C10orf114, CD55, CEACAM5, CSRP1, DND1, FHL1, IER3, ITGA6, MYEOV, SERPINE2, SLCO4A1, SSFA2, THBD, TNS4*	Increased	0.018
**GATA-1**	M00346	*CXCL5, FGFBP1, FSTL5, GPRC5A, GREM2, HAS2, HSPH1, LAMC2, POU1F1, SERPINE2, TGFA, WDR69*	Increased	0.018
**FAC1**	M00456	*A1CF, ACSM3, ALS2CR8, APOH, ARID5B, BCMO1, C14orf106, C1orf63, C20orf19, CALCOCO1, CCDC34, CCNG2, CDCA7L, CFHR1, CLMN, CRBN, CYB5A, DAB2, DIAPH2, EFNA1, EML4, ENTPD5, ERAP1, FAM111A, FAM38B, GABARAPL1, GATSL1, GLTSCR2, HBP1, HFE, HIST1H2AG, HNF4G, IFIT1, LITAF, MUT, NCAPD2, NEDD4L, NR5A2, NRG4, NRIP1, PDCD4, PECR, POF1B, SFRP4, SFRS18, SH3BGRL2, SKAP2, SLC23A2, SLC7A2, SPP1, SPTLC3, STEAP2, STX17, SYCP2L, SYNE2, TBC1D5, TBC1D8B, TGFB2, TLR1, TMC7, TMEM144, TMEM50B, VCAN, VPS13C, YPEL5, ZFYVE1, ZNF287, ZNF608*	Decreased	0.018
**SREBP-1**	M00221	*AMPD1, AQP3, CEACAM5, EREG, FGFBP1, GABRA5, GPRC5A, IL8, ITGBL1, JUN, LRRFIP1, PMEPA1, PSG8, SLCO4A1, TFPI2, THBD, TMEM171, XCL1*	Increased	0.021
**HNF4**	M01032	*ABCA1, ACSM3, AHCYL1, AKAP9, ALS2CR8, ANKRA2, ANKS4B, ANXA4, ANXA9, APOBEC3C, APOH, ARID4B, ARID5B, BCAS3, BCL2L15, BDKRB1, BDKRB2, C1RL, C4orf18, C4orf34, C5, C6orf130, CABYR, CASP4, CCDC34, CD99L2, CDH1, CEP70, CFHR1, CFHR3, CFI, CIR1, CLMN, CYHR1, CYP4F11, CYP4F12, CYP4F3, DAB2, DEPDC4, DHCR24, DNAJB4, DPYD, EFHC1, ELF3, ELP4, EPB41L4A, FAM111A, FAM55C, FGG, FKBP5, GATM, GCA, GIP, GRIP1, HAO1, HBP1, HERC6, HFE, HIST1H2AG, HMGB2, HSD17B11, ID1, IFI35, IFIT1, IFT81, KCNT2, KIAA1161, KIAA1370, KIF20A, KLHDC2, KLHL24, LHX8, LIMD1, LXN, MARCKS, MATN2, MCCC1, MEIS2, MLEC, MRAP2, MUT, NAP1L2, NBEA, NCAPD2, NDRG1, NEB, NEDD4L, NOTCH2, NOTCH2NL, NPY1R, NRG4, P2RX4, PAN2, PARP14, PARP9, PBLD, PCMTD1, PDGFC, PECR, PER2, PTGR2, RAP1GAP, RARB, RFX5, RHOBTB1, RND1, RNF213, SCNN1A, SEMA3E, SERPINB1, SESN3, SFRS18, SH3BGRL2, SLC23A2, SLC29A3, SLC35D2, SLC40A1, SLC41A2, SOAT1, SPATA7, SPP1, SPTLC3, STAT6, STRA6, SYCP2L, TBC1D5, TGFB2, TM4SF20, TMEM140, TMEM37, TMEM50B, TPCN1, TRIM31, TSKU, TTC28, TTLL6, USP3, VCAN, VPS13C, ZFYVE1, ZKSCAN1, ZNF277, ZNF287, ZNF292, ZNF608, ZNF704*	Decreased	0.023
**Pax-4**	M00377	*ACAD11, ADH6, ARMCX3, BCAS3, BTN3A1, C20orf19, C5,*	Decreased	0.023

Transcription Factor	TRANSFAC Accession Number	Predicted Gene Targets (Targets are Genes Identified as Differentially Expressed)	Targets' Expression Direction	p-value
		CACNA1D, CCDC34, CCPG1, CEACAM1, DCDC2, DEPDC6, DET1, DMXL2, ENTPD5, ERBB3, FAM105A, FNIP1, HIST1H2AG, HIST2H2BA, KIAA1712, LHX8, MIA2, NBEAL1, NR0B1, PFKFB3, PLCH1, POF1B, SASH1, SFRP4, SLC2A12, SPP1, STEAP2, TBC1D5, TFDP2, UGT2B15, WEE1, YPEL2, ZNF292, ZNF704		
**GATA-1**	M00128	*ACSS2, ADH1C, ADH6, APOBEC3C, AR, BCL2L15, BDKRB2, C5, CASP4, CCDC80, CCNG1, CDCA7L, CYFIP2, CYP4F11, CYP4F12, CYP4F3, DPYD, EFHC1, ELP4, FRK, GIP, HNF4G, KIAA1109, KIF20A, LYRM5, MYO1A, NAGA, RHOBTB3, SLC40A1, STAT6, TBC1D8B, TIGD2, TNFSF10, ZNF292*	Decreased	0.023
**Ncx**	M00484	*ABCA1, ACTA2, APAF1, C20orf194, CASP4, CDCA7L, CENPF, CEP152, CNNM2, CYB5A, CYP4F11, DIAPH2, DTX3L, EFHC1, GPRC5B, GSTA4, HBP1, ITPR2, KIAA1712, LIMA1, MANBA, NNT, NPY1R, NRG4, OSBPL9, PGAP2, PIGN, RHOBTB3, RNF213, SFRS18, SLC25A27, STEAP2, SVEP1, TMEM60, USP3, WWP1, YPEL2, ZBTB20, ZKSCAN1, ZNF704*	Decreased	0.023
**FOXP1**	M00987	*ABCA1, ABCA12, ADH1C, ADH6, ALPK1, ANKRA2, ANXA13, APH1B, AR, ARFGAP2, ARID5B, ARMCX3, ASPM, BBS9, BDH2, BNIP3L, BTN3A3, C1S, C4orf18, C5orf42, CACNA1D, CCDC28A, CCPG1, CEP152, CFH, CFI, CRBN, CTNND1, DAB2, DEPDC6, DYNC2H1, EFNA1, ELP4, EML4, ENTPD5, ERAP1, ERBB3, FAM111A, FAM149B1, FGG, FNBP1L, FNIP1, GABARAPL1, GRIP1, HAO1, HIST1H2AC, HIST2H4A, HOOK3, HSD17B11, HSD17B6, IFIT1, IFT81, INADL, ING4, ITPR2, KCNK5, KIAA1370, KIAA1632, LIMD1, LITAF, LYRM5, MANSC1, MARCKS, MCCC1, MIA2, MR1, NAP1L2, NBEA, NBEAL1, NCALD, NPY1R, NR5A2, NRG4, OSBPL9, PBLD, PCMTD1, PDCD4, PECR, PLCH1, POF1B, PTGR2, PTPLAD2, SASH1, SKAP2, SLC16A7, SLC25A27, SLC35D2, SLC41A2, SOAT1, SPATA18, SPP1, SPTLC3, STEAP2, SYCP2L, SYNE2, TBC1D5, TBC1D8B, TGFB2, THG1L, TIGD2, TLR1, TLR3, TM4SF20, TMC7, TMEM144, TMEM37, TMEM50B, TXNIP, UGT2B15, WEE1, WWP1, YPEL2, ZNF224, ZNF287, ZNF292, ZNF608, ZNF654*	Decreased	0.023
**ELF-1**	M00746	*ABCA12, ADH1C, AHCYL1, ARHGAP1, ARID4B, ARRB1, BCL2L15, BNIP3L, C1RL, C20orf74, C5orf42, CABYR, CCBL2, CCNG1, CFH, CFHR1, CTNND1, DAB2, DHCR24, DMXL2, DZIP3, EHHADH, ELMO1, ELOVL6, FGA, FRK, IQGAP2, LARGE, MARCKS, MCCC1, MR1, MUT, NAP1L2, NEK11, NR0B1, NUDT7, OPHN1, PAN2, PDK2, PGAP2, PLCD4, PLCH1, RHOBTB1, SAMD9, SESN3, SFRP4, SLC35D2, SOAT1, STAT6, TBC1D5, TGFB2, TLR1, TM4SF20, TTLL6, ZBTB20, ZNF608*	Decreased	0.026
**HNF-6**	M00639	*ABCA12, ABCG2, ADHFE1, ALDH5A1, ANO5, APOH, ARID4B, ARID5B, BLMH, BTN3A3, CACNA1D, CCBL2, CFH, DAB2, DEPDC6, DET1, DYNC2H1, EFHC1, ENTPD5, FGB, FGG, GCA, HABP2, HBP1, HIST1H2AC, HNF4A,*	Decreased	0.027

Transcription Factor	TRANSFAC Accession Number	Predicted Gene Targets (Targets are Genes Identified as Differentially Expressed)	Targets' Expression Direction	p-value
		HNF4G, KCNK5, KIAA0922, KIAA1377, KIAA1712, MARCKS, MCCC1, MTMR11, NAP1L2, NCALD, NEB, NNT, NRG4, PDZK1, PLCH1, POF1B, RFX5, SLC16A7, SLC25A27, SLC41A2, SSBP2, STX17, UGT2B15, ZNF292		
**Alx-4**	M00619	*ADH1C, BTBD11, CEP152, FGFR4, FNBP1L, HIST2H2AA3, HMGB2, HOOK3, MARCKS, NR1D2, PDCD4, PIGN, PLEKHH2, ZNF654*	Decreased	0.029
**RP58**	M00532	*AQP3, CCL2, CSRP1, DHX37, DPT, GREM2, IL8, ITGA3, ITGA6, ITGBL1, KRT38, SMOX, SNRPN, XDH*	Increased	0.034
**Lyf-1**	M00141	*ABCB6, APOH, ARSE, ATG2B, BLMH, CCBL2, CDCA7L, ERBB2, FAM111A, FAM38B, FGG, FRAS1, GRIP1, HNF4A, KIAA0922, LYRM5, MYO1A, NBEA, NRG4, PBLD, PGAP2, PLD1, PTGR2, SLC25A27, SLC40A1, SULT2B1, SYNE2, TC2N, TJP2, TSPAN15, ZKSCAN1, ZMYM3, ZNF608*	Decreased	0.036
**En-1**	M00396	*ABCA1, ACSM3, ALPK1, C1S, C20orf194, CASP4, CDCA7L, CEP152, CRBN, DIAPH2, DTX3L, EHHADH, ELOVL6, FAM105A, FAM38B, FMO5, GCA, HABP2, HIST2H4A, INADL, MANBA, MUT, NPY1R, PAIP2B, PARP14, SH3BGRL2, SLC25A27, SPTLC3, SVEP1, SYCP2L, TMEM60, UGT2B15, YPEL2, ZKSCAN1*	Decreased	0.038
**ER**	M00191	*AQP3, CLDN1, FBXO40, GABRA5, GFPT2, GLS, GPRC5A, LAMC2, LRRFIP1, P2RY4, PAQR5, PHLDA1, SEMA3C, SLCO4A1, WDR69, XDH*	Increased	0.039
**AFP1**	M00616	*ALDH5A1, ASPM, BTN3A1, C10orf57, C6orf130, CACNA1D, CALCOCO1, CCNG1, CORO2A, CTNND1, EHHADH, FGB, FOXN3, FRAS1, ID1, KCNT2, KIAA1712, KIF13B, LIMA1, LYRM5, MARCKS, MLEC, NAP1L2, NIPAL3, NIPSNAP3A, NUDT7, PBLD, PFKFB3, PLCH1, POF1B, RARB, RFX5, SFRP4, SFRS18, SLC16A7, SLC41A2, TIGD2, TLR1, TMEM144, TXNIP, UGT2B15, ZC3H6, ZMYM3, ZNF292, ZNF654*	Decreased	0.041
**Evi-1**	M00082	*ABCG2, ADH1C, ADH6, ALS2CR8, ANG, ANXA13, APOH, ASPM, BDKRB2, BNIP3L, C1orf63, C5orf42, CCNG2, CEP152, DYNC2H1, FAM55C, HIST2H2AA3, HIST2H2BA, KIAA1632, LYRM5, MANSC1, MBOAT1, MCCC1, MR1, MSI2, MYO1A, NCALD, NEB, NRG4, PIGN, PLCH1, RFX5, SAMD9, SLC41A2, SLC7A2, STAT6, TM4SF20, TMEM144, TXNDC16, TXNIP, WWP1, ZNF292, ZNF608*	Decreased	0.042
**Gfi-1**	M00250	*ACSS2, AS3MT, BCL2L11, BDH2, BLMH, CABYR, CDK5RAP3, CEP152, CTNND1, CTTNBP2, DIAPH2, ERBB3, FARP2, FGB, FGFR4, GCA, GRIP1, HBP1, HIST2H4A, HNF4A, INADL, KDM3A, KIAA1377, LIMD1, LRP1, LXN, NNT, NRG4, PAIP2B, PECR, PLCD4, POF1B, RHOBTB3, SASH1, SCAPER, SH3BGRL2, SKAP2, SLC2A12, STEAP2, TMC7, VPS39, YPEL5, ZBTB20, ZNF292*	Decreased	0.042
**LEF1**	M00805	*AKAP12, AKR1B1, ANKRD1, ANXA10, AQP3, C10orf114, CD55, CLDN1, CPA4, CPZ, CST1, CTPS, DND1, DUSP5, EMP1, EPHA2, EREG, FGFBP1, FRMD3, FSHB, FSTL5, GABRA5, GDF15, GFPT2, GLIPR1, GPRC5A, HAS2,*	Increased	0.043

Transcription Factor	TRANSFAC Accession Number	Predicted Gene Targets (Targets are Genes Identified as Differentially Expressed)	Targets' Expression Direction	p-value
		HBEGF, HTR3D, IL8, ITGA3, ITGBL1, KIAA1199, KRT38, KRT80, LAMC2, NAV3, NOSTRIN, NRG1, ODC1, PMEPA1, POU1F1, PSG8, RAB3B, S100A3, SAMD7, SEMA3C, SERPINB8, SLCO4A1, SOCS2, SPRR2B, STC1, THBD, TNS4, UBASH3B, XCL1, XDH		
**OTX**	M01117	*ANKRA2, C14orf106, CASP6, CRBN, CTNND1, CTTNBP2, DIAPH2, ELF3, FAM149B1, FGB, HIST1H2AG, HIST2H4A, IP6K2, KDM3A, KLHDC2, MARCKS, NFKBIZ, NIPSNAP3A, NUDT7, PCDH9, PCMTD1, PECR, PLCD4, SESN3, TBCK, TSPAN15, WEE1, ZNF292*	Decreased	0.047
**CDP**	M00102	*ABCA1, ACAD11, ALPK1, ALS2CR8, APOH, ARFGAP2, ARID5B, ARMCX3, BAMBI, BCMO1, BNIP3L, BTN3A3, C4orf34, CCBL2, DAB2, DEPDC6, DET1, DYNC2H1, DZIP3, EFHC1, FGB, FGG, HIST1H2AG, HIST2H4A, HNF4A, HNF4G, HOXA2, INADL, KIAA1109, MARCKS, MCCC1, MTMR11, NIPSNAP3A, NRG4, NUSAP1, PDGFC, PDZK1, PIGN, SASH1, SEMA3E, SERPINA6, SFRS18, SLC16A7, SLC41A2, STEAP2, STX17, TBC1D5, TBCK, TLR3, TM4SF20, TMEM136, TXNDC16, UGT2B15, ZNF654*	Decreased	0.047
**c-Ets-1**	M00339	*ANXA3, CALB1, CD55, CSRP1, CXCL5, DUSP1, LAMC2, LMO7, NCEH1, NMNAT2, NOSTRIN, NR4A1, NRG1, PHLDA1, PLEK2, PSG8, S100A3, SOCS2, SSFA2, TGFA, THBD, TMEM171, TNS4*	Increased	0.049

Supplemental Material, Table 6: Genes commonly differentially expressed upon exposure to photochemically altered (PCA) pollutants or cigarette smoke (CS).

Genes identified as differentially expressed due to PCA exposure that are also differentially expressed in human lung cells exposed to cigarette smoke (Maunders et al. 2007) are listed, along with their direction of gene expression fold change (FC).

Gene Symbol	PCA Pollutant-Induced FC Direction, 9 hr Post-Exposure	CS-Induced FC Direction, 1 hr Post-Exposure	CS-Induced FC Direction, 6 hr Post-Exposure	CS-Induced FC Direction, 24 hr Post-Exposure
ALDH5A1	decrease	decrease	decrease	decrease
ALDH6A1	decrease	decrease	decrease	decrease
ATP8B1	decrease	decrease	decrease	decrease
BMPR2	decrease	decrease	decrease	decrease
C1RL	decrease	decrease	decrease	decrease
CDH1	decrease	decrease	decrease	decrease
DTX3L	decrease	decrease	decrease	decrease
EIF2C4	decrease	decrease	decrease	decrease
EML4	decrease	decrease	decrease	decrease
ERBB3	decrease	decrease	decrease	decrease
FCHSD2	decrease	decrease	decrease	decrease
HOOK1	decrease	decrease	decrease	decrease
KIAA1161	decrease	decrease	decrease	decrease
KIAA1377	decrease	decrease	decrease	decrease
LBA1	decrease	decrease	decrease	decrease
MARCKS	decrease	decrease	decrease	decrease
NCOA2	decrease	decrease	decrease	decrease
NFIA	decrease	decrease	decrease	decrease
NOTCH2	decrease	decrease	decrease	decrease
PARP14	decrease	decrease	decrease	decrease
PDPR	decrease	decrease	decrease	decrease
RBPMS	decrease	decrease	decrease	decrease
ROBO1	decrease	decrease	decrease	decrease
SASH1	decrease	decrease	decrease	decrease
SESN3	decrease	decrease	decrease	decrease
SLC7A2	decrease	decrease	decrease	decrease
STX17	decrease	decrease	decrease	decrease
SYNE2	decrease	decrease	decrease	decrease
TGFB2	decrease	decrease	decrease	decrease
TP53INP1	decrease	decrease	decrease	decrease
VPS13C	decrease	decrease	decrease	decrease
ZKSCAN1	decrease	decrease	decrease	decrease
MSI2	decrease	decrease	decrease	increase
NOTCH2NL	decrease	decrease	decrease	increase
FBXO32	decrease	decrease	increase	decrease
TFDP2	decrease	decrease	increase	decrease

Gene Symbol	PCA Pollutant-Induced FC Direction, 9 hr Post-Exposure	CS-Induced FC Direction, 1 hr Post-Exposure	CS-Induced FC Direction, 6 hr Post-Exposure	CS-Induced FC Direction, 24 hr Post-Exposure
WSB1	decrease	decrease	increase	decrease
INADL	decrease	decrease	increase	increase
PCDH9	decrease	decrease	increase	increase
CLDN1	increase	decrease	decrease	decrease
GLIPR1	increase	decrease	decrease	increase
LRRFIP1	increase	decrease	decrease	increase
SSFA2	increase	decrease	decrease	increase
AREG	increase	decrease	increase	increase
DUSP4	increase	decrease	increase	increase
DUSP5	increase	decrease	increase	increase
EREG	increase	decrease	increase	increase
GDF15	increase	decrease	increase	increase
HBEGF	increase	decrease	increase	increase
JUN	increase	decrease	increase	increase
NR4A1	increase	decrease	increase	increase
PHLDA1	increase	decrease	increase	increase

REFERENCES

Dennis Jr G, Sherman BT, Hosack DA, Yang J, Baseler MW, Lane HC, et al. 2003. DAVID: Database for Annotation, Visualization, and Integrated Discovery. Genome Biology 4:P3.

g:Profiler. 2011. BIIT Group, Institute of Computer Science. Available: http://biit.cs.ut.ee/gprofiler [Accessed 11 April 2011].

Hosack DA, Dennis Jr G, Sherman BT, Lane HC, Lempicki RA. 2003. Identifying biological themes within lists of genes with EASE. Genome Biology 4:R70.

Huang DW, Sherman BT, Lempicki RA. 2009a. Bioinformatics enrichment tools: paths toward the comprehensive functional analysis of large gene lists. Nucleic Acids Res 37:1-13.

Huang DW, Sherman BT, Lempicki RA. 2009b. Systematic and integrative analysis of large gene lists using DAVID bioinformatics resources. Nature Protocols 4:44-57.

Maunders H, Patwardhan S, Phillips J, Clack A, Richter A. 2007. Human bronchial epithelial cell transcriptome: gene expression changes following acute exposure to whole cigarette smoke in vitro. Am J Physiol Lung Cell Mol Physiol 292:L1248-1256.

Reimand J, Kull M, Peterson H, Hansen J, Vilo J. 2007. g:Profiler - a web-based toolset for functional profiling of gene lists from large-scale experiments. Nucleic Acids Res 35:W193-200.
